# Ambient long-term exposure to organophosphorus pesticides and the human gut microbiome: an observational study

**DOI:** 10.1186/s12940-024-01078-y

**Published:** 2024-04-16

**Authors:** Keren Zhang, Kimberly Paul, Jonathan P. Jacobs, Myles G. Cockburn, Jeff M. Bronstein, Irish del Rosario, Beate Ritz

**Affiliations:** 1 Department of Epidemiology, UCLA Fielding School of Public Health, Los Angeles, CA USA; 2 Department of Neurology, UCLA David Geffen School of Medicine, Los Angeles, CA USA; 3 The Vatche and Tamar Manoukian Division of Digestive Diseases, Department of Medicine, David Geffen School of Medicine at UCLA, Los Angeles, CA USA; 4 Division of Gastroenterology, Hepatology and Parenteral Nutrition, VA Greater Los Angeles Healthcare System, Los Angeles, CA USA; 5 Department of Population and Public Health Sciences, Keck School of Medicine, University of Southern California, Los Angeles, CA USA; 6 Department of Environmental Health Sciences, UCLA Fielding School of Public Health, Los Angeles, CA USA

**Keywords:** Organophosphorus pesticides, Gut microbiome, Predicted metagenome, Geographic information system

## Abstract

**Background:**

Organophosphorus pesticides (OP) have been associated with various human health conditions. Animal experiments and in-vitro models suggested that OP may also affect the gut microbiota. We examined associations between ambient chronic exposure to OP and gut microbial changes in humans.

**Methods:**

We recruited 190 participants from a community-based epidemiologic study of Parkinson’s disease living in a region known for heavy agricultural pesticide use in California. Of these, 61% of participants had Parkinson’s disease and their mean age was 72 years. Microbiome and predicted metagenome data were generated by 16S rRNA gene sequencing of fecal samples. Ambient long-term OP exposures were assessed using pesticide application records combined with residential addresses in a geographic information system. We examined gut microbiome differences due to OP exposures, specifically differences in microbial diversity based on the Shannon index and Bray–Curtis dissimilarities, and differential taxa abundance and predicted Metacyc pathway expression relying on regression models and adjusting for potential confounders.

**Results:**

OP exposure was not associated with alpha or beta diversity of the gut microbiome. However, the predicted metagenome was sparser and less evenly expressed among those highly exposed to OP (*p* = 0.04). Additionally, we found that the abundance of two bacterial families, 22 genera, and the predicted expression of 34 Metacyc pathways were associated with long-term OP exposure. These pathways included perturbed processes related to cellular respiration, increased biosynthesis and degradation of compounds related to bacterial wall structure, increased biosynthesis of RNA/DNA precursors, and decreased synthesis of Vitamin B1 and B6.

**Conclusion:**

In support of previous animal studies and in-vitro findings, our results suggest that ambient chronic OP pesticide exposure alters gut microbiome composition and its predicted metabolism in humans.

**Supplementary Information:**

The online version contains supplementary material available at 10.1186/s12940-024-01078-y.

## Background

The microbiome refers to the collective genome of all microorganisms (bacteria, viruses, archaea, fungi, etc.) colonizing the human body [1, 2]. The gut microbiome has drawn special attention in recent decades, as it plays important roles in human health and pathology [3, 4] as well as in metabolism, including energy intake, synthesis and absorption of nutrients, and detoxification [5, 6]. The gut microbiome also plays a major role in the regulation of immune defenses [7, 8]. Recent animal studies have indicated that environmental exposures such as pesticides, air pollution, and heavy metals can influence the human gut microbiome and the immune system [9–12]. To date, human evidence is still sparse but efforts to link environmental factors to the human microbiome are growing. Furthermore, the gut microbiome may have the potential to serve as a crucial link between environmental exposure and development of neurological diseases via the gut-brain axis [13]. Bidirectional connections between the gut microbiome and the brain are supported by the actions and functions of the autonomous nervous system, the endocrine and immune systems. The growing recognition that the gut and the brain interact has inspired new avenues of research linking environmental exposures to the gut microbiome [14]. Gut dysbiosis presents an imbalance in microbial composition that can alter human metabolism. In addition to lifestyle factors (e.g., diet, physical activity), ubiquitous toxicants in the human environment may contribute to such an imbalance.

Organophosphorus pesticides (OP), commonly used as insecticides and nematicides, are toxicants of interest as their metabolites have been found in a majority (60 percent or more) of urine samples of participants in NHANES 2011–2012, 2015–2016, and 2017–2018, a multi-wave nationwide representative sample of the US population [15]. While acute toxicity of OP to humans is well established [16], effects of chronic low-level exposure remain less explored [2, 17]. Thus far, chronic OP exposures have been suggested to be involved in metabolic conditions such as obesity, glucose intolerance, and diabetes [18], neurological conditions including Parkinson’s disease [19], Alzheimer’s disease [20, 21], and some chronic mental disorders such as depression and anxiety [22].

The current developments in microbiome research have enabled us to assess potential dysbiosis by evaluating the biodiversity of gut microorganisms [23], as well as discover associations between bacterial groups (e.g., phyla, families, genera, etc.) and exposure or disease status through differential abundance analysis [24]. Additionally, predicted metagenomic data reflecting potential metabolic functions allow us to generate hypotheses about the possible biological mechanisms underlying interactions between the environment, the gut microbiome, and the human host’s health [25]. Here, we explore whether ambient long-term exposure to OP pesticides may alter the human gut microbiome composition and/or function in a community-based epidemiological study in rural California. We relied on a geographic information systems (GIS)-based approach to link pesticide application records with participant addresses and estimated ambient pesticide exposure levels.

## Methods

### Study population and sample collection

This study relies on data collected in the Parkinson’s, Environment and Gene study (PEG), a population-based, case–control study conducted in Kern, Tulare and Fresno counties of California. It was initially designed to investigate the etiology of Parkinson’s disease (PD) and participants were recruited in two study waves: 2001–2007 and 2012–2017. At baseline, PD patients were diagnosed within the past 5 years and randomly selected community controls were also recruited. Since 2017, we invited previous study participants who could be contacted to enroll in a pilot study of the gut microbiome. In addition, we invited a household or community member of PD patients to participate. All participants were asked to submit a fecal sample and allow us to conduct standardized interviews if they met the following eligibility criteria: they did not have 1) acute/chronic gastrointestinal conditions; or 2) an immunocompromised state and/or were taking immunosuppressants; 3) antibiotic intake continuously or within the past three months. In total 190 participants with complete data were included in the study. This study was approved by the UCLA Institutional Review Board. Informed written consent was obtained from all study participants.

Trained research staff conducted structured interviews with the participants and recorded information for demographics and lifetime occupational and residential histories. Each participant was provided with a standardized self-collection kit that included all materials needed for fecal sample collection and also detailed instructions. The collection kit was assembled by our study research staff based on the protocol developed by the UCLA Microbiome Core (currently part of the Microbiome Core of the Goodman-Luskin Microbiome Center) [26]. Briefly, fecal samples were collected by participants at home and preserved in sterilized ParaPak® vial with 96% ethanol. Samples were received within 14 days of collection and stored at -80 °C degrees until DNA extraction and sequencing. Studies of stool preservation methods have consistently found that self-collected stool immediately preserved in ethanol will give similar results as the gold standard of immediately fresh frozen stool [27–29].

### Microbiome assessment and metagenomic prediction

The ZymoBIOMICS DNA kit was used to extract bacterial DNA from fecal samples with bead beating. The V4 region of 16s rRNA genes was amplified and underwent pair-ended 250 × 2 sequencing on Illumina platforms (HiSeq 2500 or MiSeq). Raw sequencing data were obtained and processed using the DADA2 pipeline (v1.22.0) and phyloseq package (v1.34.0) in R (v4). Sequencing reads were quality-filtered and processed into amplicon sequence variants (ASVs); a classification that corresponds to species level taxonomy. ASVs were further assigned to corresponding taxonomy by closed-reference picking against the Silva v132 database [30]. ASVs were further filtered by taxonomy in two steps: first by total abundance—ASVs were removed if the phyla they belong to have a total abundance of less than 50, then by prevalence—ASVs were removed if prevalence was less than 10% in all samples. The sequencing depths ranged from 5,365 to 65,499 with a mean depth of 32,637 $\pm$ 11,879 per sample after the filtering steps, which corresponds to 410 ASVs, 147 genera, 49 families, and 10 phyla. ASVs were also rarefied to even depth without abundance/prevalence filtering to assess alpha diversity. The relative abundance of bacterial taxa for the study sample are shown in Figure S1 and S2.

PICRUST2 (v2.4.1) was used to predict the functional potential of the bacterial community based on 16s rRNA marker sequencing data, i.e., the metagenomic profile of the gut microbiome [31]. Based on the 16s marker gene sequences, the gene contents of the gut microbes, classified by enzyme commission (EC) number, and its metabolic functions, i.e., Metacyc pathway profiles were predicted. Predicted bacterial genes and pathways were removed if the total abundance was less than 100, or the prevalence was less than 10% in study samples. 1,823 EC and 363 Metacyc pathways remained after the abundance/prevalence filtering step.

### Pesticide exposure assessment

We used a GIS method to estimate the ambient pesticide exposure of each study participant. With this method, we linked data from three sources: (1) California Pesticide Use Reports (PUR), a mandatory commercial pesticide application reporting system collecting data since 1974; (2) land-use survey data (based on California’s Public Land Survey System), which provide the exact location of specific crops; and (3) lifetime residential history reported by participants, which provides the location of and duration at the participants’ residence. We generated the annual poundage of every reported pesticide applied within 500-m of a participant’s residential address relying on their lifelong self-reported address histories. A 500-m buffer was selected based on evidence from studies measuring deposits of ground and aerial pesticide applications [32, 33], California studies of pesticide residues measured inside homes in proximity to agricultural applications [34, 35], and to maintain consistency with previously published work from the PEG study [36]. Our main analyses were based on a 10-year exposure period prior to fecal sample collection to represent a plausible window for chronic effects of pesticides on the gut microbiome. Thus, we calculated 10-year average exposures from the annual measures prior to fecal sample collection. In sensitivity analyses, we additionally explored two alternative exposure periods, one 5-year window with and one without a 5-year lag, i.e., the period 6–10 years or 0–5-years priors to sample collection, respectively.

Here, we focus on the 36 chemicals categorized as OP from the PUR records (Table 1). As the toxicity of exposure per pound applied varies across different OP chemicals, we first evaluated each OP pesticide separately. For each specific OP pesticide, we considered a participant exposed if their annual averaged exposure poundage was higher than the median poundage observed in non-PD participants. The dichotomized OP exposure values (yes/no) for all 36 OP were then summed to derive the overall OP exposure (theoretically ranging from 0 to 36). Finally, based on all 36 individual chemicals, we categorized participants into high OP exposure and low/no OP exposure: participants who were exposed to > 1 chemical were considered highly exposed and those with exposure to ≤ 1 made up the reference group. Participants’ exposure to other pesticide groups other than OP were combined into a summary estimate (ever/never) based on five major pesticide use types: fumigants, herbicides, fungicides, insecticides, and fungicides/insecticides (pesticides used as both fungicides and/or insecticides). Briefly, covering the same exposure period as the OP exposure measures, we generated exposure categories for other pesticide groups. Ever/never instead of a high/low category was used as the exposure prevalence was generally low in these periods. Participants thus are considered “exposed to other pesticides beside OPs” if they were exposed to any of the five other pesticide groups.

**Table 1:** List of organophosphorus pesticides of interest

Acephate	Merphos
Azinphos-Methyl	Methamidophos
Bensulide	Methidathion
Carbophenothion	Methyl Parathion
Chlorpyrifos	Mevinphos
Ddvp	Monocrotophos
Demeton	Naled
Dialifor	Oxydemeton-Methyl
Diazinon	Parathion
Dicrotophos	Phorate
Dimethoate	Phosalone
Dioxathion	Phosmet
Disulfoton	Phosphamidon
Ethephon	Profenofos
Ethion	S,S,S-Tributyl Phosphorotrithioate
Fenamiphos	Sulfotep
Leptophos	Tepp
Malathion	Trichlorfon

### Statistical analysis

We examined the global microbiome profile for alpha diversity (Shannon index) and beta diversity (Bray–Curtis dissimilarity). The mean differences in alpha diversity between exposure groups was assessed using the Wilcoxon test statistic and multivariable linear regression, while exposure group-based beta diversity differences were visually examined using plots from Principal Coordinate Analysis and tested with permutation multivariate analysis of variance (PERMANOVA).

Taxa abundance was compared between exposure groups at genus, family, and phylum levels separately. Using MaAsLin2, an R package designed for univariate differential taxa abundance analysis, we assumed a negative binomial distribution and normalized our microbiome data using the trimmed mean method [37]. We adjusted for sex, age, race (white vs non-white), PD status (PD vs non-PD), exposure to other pesticide groups (fumigants, herbicides, fungicides, and insecticides other than OP, Table S1), and sequencing platform (HiSeq vs MiSeq) in the regression model to control for potential confounding. We used Benjamini–Hochberg corrections to control for false discovery rate at < 0.05.

Similarly, we explored associations between predicted metagenomic data and pesticide exposure using the Wilcoxon test to assess differences in gene richness (i.e. Shannon diversity of predicted bacterial functional genes within each sample), the PERMANOVA test to assess differences in beta diversity (Bray–Curtis dissimilarity of the gene count between samples), and MaAslin2 to perform regression modeling for differential abundance of Metacyc pathways by pesticide exposure status, controlling at a minimum for race, sex, age, PD status, co-exposure to other pesticide groups and sequencing platform. The analyses described above were performed with SAS 9.4 and R (v4).

We conducted sensitivity analyses to assess potential bias arising from exposure misclassifications and confounding by PD status. First, to examine the changes in microbial and predicted metabolic pathways related to change of exposure window, we employed two alternative windows—exposure to OP during 6–10 years (Sensitivity Model 1) and 0–5 years (Sensitivity Model 2) prior to sample collection (Figure S3). 19 participants previously considered highly exposed were moved into the low exposure group in the 0–5-year window, whereas in the 6–10-year window prior to samples collection, only 2 participants changed exposure status compared to the entire 10-year window. Second, to assess whether PD status confounded the associations, we compared effect estimates from models that did and did not control for PD status (Sensitivity Model 3).

## Results

The 190 participants with a fecal sample and complete demographic information in the analysis (Table 2), were on average 72 years old, 53% were males (*N* = 101), 74% were white (*N* = 140), 61% (*N* = 116) had PD, and 36 participants (19%) were considered highly exposed to OP pesticides at their residences within the 10-year period prior to fecal sample collection. The distribution of OP exposure is shown in Figure S4.

**Table 2:** Demographics and exposure to pesticides of the study population (*N* = 190)

Minority	
White	140 (73.7%)
Non-white	50 (26.3%)
Sex	
Male	101 (53.2%)
Female	89 (46.8%)
Age at sample collection	
Mean (SD)	72.1 (8.73)
Median [Min, Max]	72.5 [43.0, 95.0]
Parkinson's Disease (PD) status	
Non-PD	74 (38.9%)
PD	116 (61.1%)
Sequencing platform	
HiSeq	141 (74.2%)
MiSeq	49 (25.8%)
Exposure to organophosphates	
Low Exposure	154 (81.1%)
High Exposure	36 (18.9%)
Exposure to other pesticide groups	
Low Exposure	115 (60.5%)
High Exposure	75 (39.5%)

Crude comparisons of alpha (Shannon index) and beta diversity (Bray–Curtis dissimilarity) showed no differences in bacterial diversity with OP exposure (Figure S5). Similarly, neither multivariable linear regression analysis using the Shannon index and adjusting for potential confounders (*p* = 0.31) nor the PERMANOVA test for difference in bacterial composition (*p* = 0.64) showed associations with OP exposures. However, differential abundance analyses found 13 genera and one family that exhibited positive associations with OP exposure, while 9 genera and one family were negatively associated with OP exposure. The majority of the differentially abundant genera belong to the family of Lachnospiraceae (9 genera) and Ruminococcaceae (7 genera) within the Clostridia class, with the remainder belonging to the family of Burkholderiaceae, Erysipelotrichaceae, Clostridiales_vadinBB60_group, Acidaminococcaceae, Synergistaceae, and Veillonellaceae (Table 3, Table S2-S4, Figure S6).

**Table 3:** Differential taxa abundance associated with organophosphorus pesticides (*N* = 190)

Phylum	Family	Genus	Log2FC	SE	Adj *P*
Bacteroidetes	Barnesiellaceae	–	0.59	0.05	8.739E-35
Actinobacteria	Coriobacteriales_Incertae_Sedis	–	-1.02	0.08	4.644E-35
Firmicutes	Lachnospiraceae	*Sellimonas*	0.94	0.04	1.908E-113
Proteobacteria	Burkholderiaceae	*Sutterella*	0.59	0.03	1.621E-69
Firmicutes	Lachnospiraceae	*Blautia*	0.53	0.17	6.613E-03
Firmicutes	Ruminococcaceae	*Ruminococcaceae_UCG-014*	0.40	0.04	3.839E-19
Firmicutes	Lachnospiraceae	*UC5-1-2E3*	0.38	0.05	6.201E-14
Firmicutes	Ruminococcaceae	*Ruminococcaceae_UCG-010*	0.34	0.06	7.809E-07
Firmicutes	Lachnospiraceae	*Lachnospiraceae_UCG-004*	0.32	0.05	4.073E-10
Firmicutes	Lachnospiraceae	*Tyzzerella_4*	0.31	0.04	1.174E-16
Firmicutes	Clostridiales_vadinBB60_group	*Unspecified*	0.28	0.06	2.469E-06
Firmicutes	Lachnospiraceae	*Coprococcus_1*	0.23	0.02	4.847E-20
Firmicutes	Lachnospiraceae	*CAG-56*	0.20	0.04	3.477E-07
Firmicutes	Ruminococcaceae	*Ruminococcaceae_UCG-004*	0.11	0.03	7.231E-04
Firmicutes	Acidaminococcaceae	*Acidaminococcus*	0.07	0.02	4.827E-03
Firmicutes	Lachnospiraceae	*Lachnospiraceae_FCS020_group*	-0.15	0.06	3.938E-02
Firmicutes	Veillonellaceae	*Dialister*	-0.17	0.02	1.383E-20
Firmicutes	Lachnospiraceae	*Tyzzerella*	-0.20	0.06	4.041E-03
Firmicutes	Ruminococcaceae	*DTU089*	-0.35	0.06	3.874E-09
Firmicutes	Erysipelotrichaceae	*Holdemania*	-0.43	0.12	1.088E-03
Firmicutes	Ruminococcaceae	*Anaerotruncus*	-0.63	0.11	8.867E-08
Firmicutes	Erysipelotrichaceae	*Turicibacter*	-1.54	0.61	4.351E-02
Proteobacteria	Burkholderiaceae	*Parasutterella*	-2.13	0.49	6.251E-05
Synergistetes	Synergistaceae	*Cloacibacillus*	-2.47	0.93	3.179E-02

Model was adjusted for sex, minority status, age, Parkinson’s disease status, pesticide co-exposure, and sequencing platform

*Abbreviations Log2FC*, Log2 Fold Change, *SE* Standard Error, *Adj* Adjusted

According to the Shannon index, the richness and evenness of bacterial genes predicted from gut microbiota composition was marginally higher in participants exposed to fewer OP than in participants considered highly exposed (Figure S7, *p* = 0.04). The composition of the metagenome, however, was not associated with OP exposure (PERMANOVA, *p* = 0.28). Overall, 20 pathways were predicted to have increased expression and 14 pathways exhibited decreased expression with higher chronic OP exposure (Table 4, Table S5, Figure S8-S9). These pathways belong to the superclasses of: 1) Cofactor, prosthetic group, electron carrier, and vitamin biosynthesis; 2) Nucleoside and nucleotide biosynthesis and degradation; 3) Carbohydrate biosynthesis and degradation; 4) Amino acid biosynthesis and degradation; 5) Cell structure biosynthesis; 6) Fermentation; 7) Amine and polyamine degradation; 8) C1 compound utilization and assimilation 9) Cell structure biosynthesis; 10) Polymeric compound degradation; 11) Respiration; 12) Secondary metabolite biosynthesis; and 13) Superpathways of histidine, purine, and pyrimidine biosynthesis.

**Table 4:** Differential predicted Metacyc pathways abundance associated organophosphorus pesticides (*N* = 190)

Metacyc Pathway	Log2 FC	SE	Adj *P*
Pyrimidine deoxyribonucleotides de novo biosynthesis IV	0.40	0.12	1.626E-02
Methanogenesis from acetate	0.40	0.12	9.864E-03
Pyrimidine deoxyribonucleotides biosynthesis from CTP	0.37	0.11	1.513E-02
Teichoic acid (poly-glycerol) biosynthesis	0.37	0.09	2.158E-03
dTDP-N-acetylthomosamine biosynthesis	0.36	0.12	3.302E-02
Fucose degradation	0.33	0.09	3.398E-03
L-valine degradation I	0.26	0.06	1.046E-04
Superpathway of N-acetylglucosamine, N-acetylmannosamine and N-acetylneuraminate degradation	0.26	0.06	1.150E-03
Superpathway of L-alanine biosynthesis	0.24	0.08	3.529E-02
Sucrose degradation III (sucrose invertase)	0.21	0.06	1.843E-02
Acetylene degradation	0.20	0.06	2.007E-02
Peptidoglycan maturation (meso-diaminopimelate containing)	0.20	0.07	3.053E-02
Superpathway of pyrimidine deoxyribonucleosides degradation	0.19	0.05	8.221E-03
Superpathway of purine deoxyribonucleosides degradation	0.19	0.06	1.909E-02
Purine ribonucleosides degradation	0.17	0.06	4.512E-02
L-lysine biosynthesis I	0.14	0.04	1.453E-02
Galactose degradation I (Leloir pathway)	0.13	0.04	3.328E-02
Pyruvate fermentation to isobutanol (engineered)	0.11	0.04	3.302E-02
L-lysine biosynthesis VI	0.10	0.03	4.718E-02
Calvin-Benson-Bassham cycle	0.09	0.03	4.892E-02
Superpathway of thiamin diphosphate biosynthesis II	-0.23	0.08	3.390E-02
Chitin derivatives degradation	-0.34	0.04	4.191E-14
Creatinine degradation II	-0.36	0.05	1.233E-12
Superpathway of histidine, purine, and pyrimidine biosynthesis	-0.37	0.11	1.215E-02
Superpathway of pyridoxal 5'-phosphate biosynthesis and salvage	-0.39	0.13	3.378E-02
Pyridoxal 5'-phosphate biosynthesis I	-0.41	0.14	3.959E-02
Isoprene biosynthesis II (engineered)	-0.45	0.02	2.081E-146
4-aminobutanoate degradation V	-0.51	0.14	5.027E-03
Ubiquinol-7 biosynthesis (prokaryotic)	-0.71	0.25	4.830E-02
Ubiquinol-9 biosynthesis (prokaryotic)	-0.71	0.25	4.830E-02
Ubiquinol-10 biosynthesis (prokaryotic)	-0.71	0.25	4.830E-02
Ubiquinol-8 biosynthesis (prokaryotic)	-0.71	0.25	4.830E-02
Superpathway of ubiquinol-8 biosynthesis (prokaryotic)	-0.72	0.25	4.505E-02
Formaldehyde assimilation I (serine pathway)	-0.94	0.04	1.613E-132

Model was adjusted for sex, minority status, age, Parkinson's disease status, pesticide co-exposure, and sequencing platform

*Abbreviations*: *Log2FC* Log2 Fold Change, *SE* Standard Error, *Adj* Adjusted

In analyses using the 6–10 year window prior to sample collection (Sensitivity Model 1), all observed associations for bacterial abundance remained very similar, except for the Coriobacteriales_Incertae_Sedis family and six genera for which differences were no longer formally statistically significant (alpha = 0.05). For the 0–5 year window prior to sample collection (Sensitivity Model 2), results were more varied and only six genera we identified overlapped with the full 10-year exposure window. When excluding PD status from the regression models, all associations remained, except that for the *Sellimonas* and the *Turicibacter* genus associations that were no longer formally statistically significant (Table S2-S4). In predicted Metacyc pathway expression analyses, all associations remained consistent in direction and effect size, except for L-valine degradation I, for which there was no association seen in the 6–10 year exposure window (Table S5).

## Discussion

We explored associations between long-term ambient OP exposure from agricultural applications and the human gut microbiome. The ultimate goal was to assess whether pesticide exposures affect human neurodegenerative diseases via the gut microbiome, its metabolome, or its influence on immune function. Viewing the gut microbiome as an ecosystem, we did not observe changes in bacterial diversity or general composition with OP exposures. This is not unexpected as the exposures we modeled are chronic and low dose, and inherent resilience allows the gut microbiome to return to an equilibrium following even major perturbations such as antibiotic treatment [38]. Therefore, dramatic changes in microbial load or diversity that have been observed in acute exposure scenarios used in animal experiments are unlikely to be replicated in humans who are not acutely pesticide poisoned.

However, at a higher resolution, we found differences in the abundance of individual bacterial taxa at the family and genus level. At the family level, we observed an increase of Barnesiellaceae and decrease of Coriobacteriales Incertae Sedis with exposure to OP. Barnesiellaceae have been linked to chronic conditions such as obesity [39], cognitive dysfunction [40], depression [41], cardiovascular disease [42], and Crohn’s disease [43]. Coriobacteriales Incertae Sedis is a family of Coriobacteriia of uncertain origin identified in various environments including soil, water, as well as the animal and human gut. Both bacterial families have not been associated with OP exposure previously, and the observed associations appear less consistent in our sensitivity analysis, indicating that they may have been due to chance. Further study is necessary to understand their impact on human health.

Most abundance changes at the genus level associated with high ambient OP exposure belong to the Lachnospiraceae (seven genera were increased and two were decreased) and Ruminococcaceae (3 genera were increased and 2 were decreased) families in the Clostridia class. Lachnospiraceae and Ruminococcaceae are two groups of closely related strictly anaerobic bacteria that dwell in the gut of healthy individuals. Although some species or genera within the family are linked to certain diseases [44–46], Lachnospiraceae and Ruminococcaceae are often considered beneficial bacteria as they produce short-chain fatty acids (SCFAs) via fermentation of dietary fibers [47, 48]. SCFAs mostly refer to acetate, propionate, and butyrate, metabolites which are critical in maintaining the homeostasis of the gut microbiome including gut barrier integrity, immunomodulation and regulation of the metabolism of lipids, cholesterol, and glucose [48–50]. The production of SCFAs is determined by the type of dietary fibers, the fermenting bacteria, the gut environment, and the substrate [51]. Therefore, it is possible that the observed changes in SCFA-producing bacteria are an indicator of disturbed homeostasis of the gut environment due to chronic OP exposure, and the body’s response to such changes. Interestingly, the production of SCFAs, specifically acetate, has been observed during bacterial degradation of OP pesticides [52, 53]. Acetate has been shown to enhance glycogen repletion in liver and skeletal muscle and to account for glucose intolerance [54, 55]. Thus, as OP exposures have previously been reported to be associated with some metabolic conditions such as obesity these effects may be mediated by the gut microbiome, more specifically via the production and function of SCFAs.

Based on the predicted metagenome of gut microbiota, lower bacterial gene diversity and altered relative abundance of several metabolic pathways were linked to higher OP exposure. This suggests an alteration of the gut environment that generally leads to a decrease in expressed genetic abundance and related metabolic function. In addition, our predicted metagenomic pathway data indicated increased methanogenesis from acetate, which reflects the expected increased production of acetate metabolites from OPs.

**Fig. 1:**
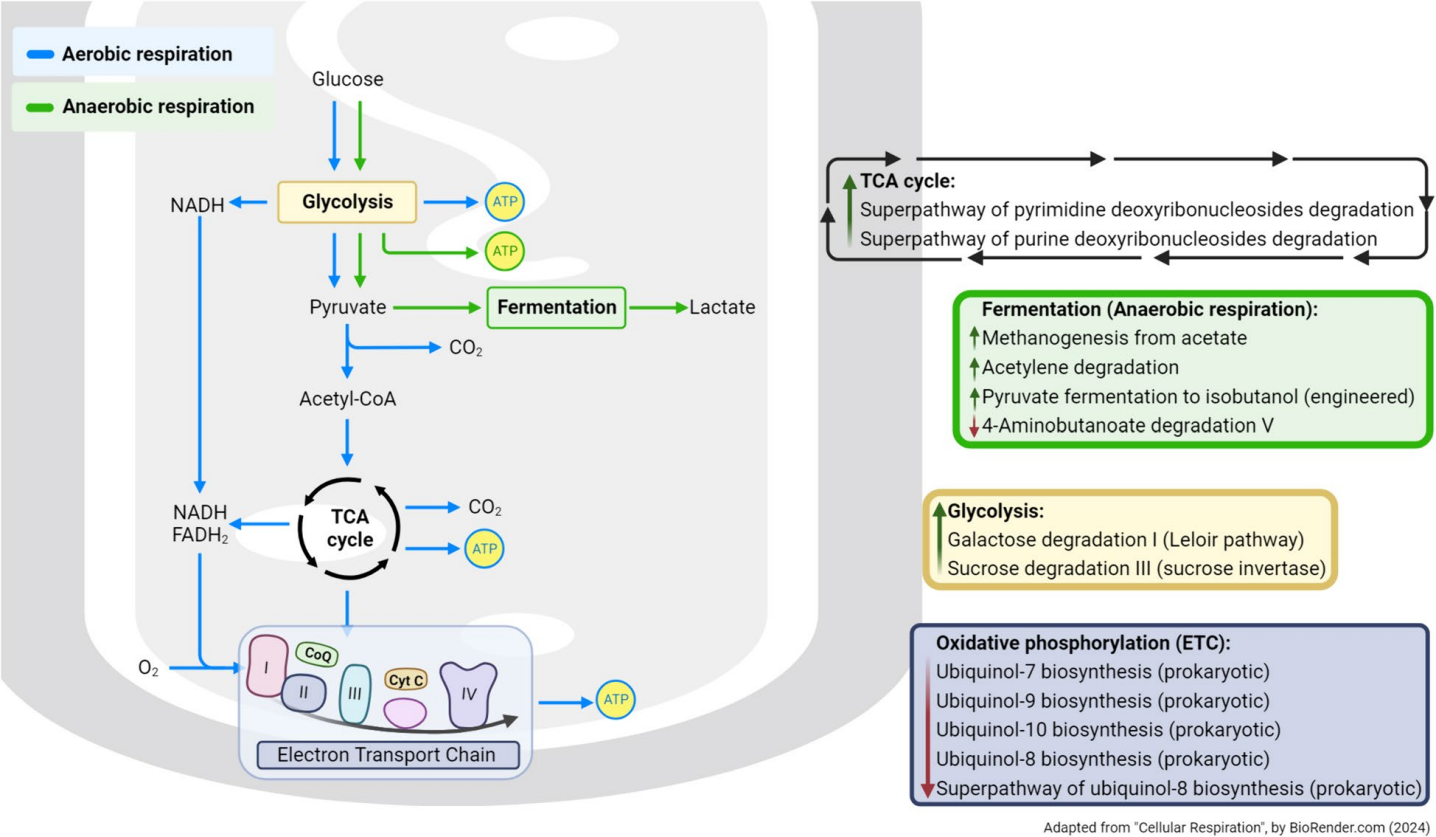
Predicted respiration pathways perturbed by ambient organophosphorus pesticides exposure

Interestingly, we noticed that a series of pathways affected by OP exposure were related to cellular respiration and subsequent energy production (Fig. 1), such as processes involved in glycolysis, the citric acid cycle, and electron transport chain, resembling the aerobic respiration in the mitochondria of eukaryotes. OPs have been shown to induce cellular oxidative stress and impair enzymatic pathways involved in metabolism of carbohydrates, fats and protein within the cytoplasm, mitochondria, and peroxisomes [56]. Mitochondria have previously been proposed as potential targets of OP pesticides [57]. Mitochondrial symbiosis, i.e. prokaryotes taken inside another through endosymbiosis, is believed to be the genesis of eukaryotes [58]. Therefore, the predicted metagenomic change in gut microbiota of chronically OP exposed individuals provides a unique perspective on OP mitochondrial toxicity in humans. OPs seem to affect the gut microbiome mitochondrial metabolism similar to human cell mitochondrial function. In addition, OP exposure is predicted to affect a number of fermentation processes in microbes, which may also affect the production of energy and precursor metabolites for downstream pathways, altering the metabolic activity of microbes.

**Fig. 2:**
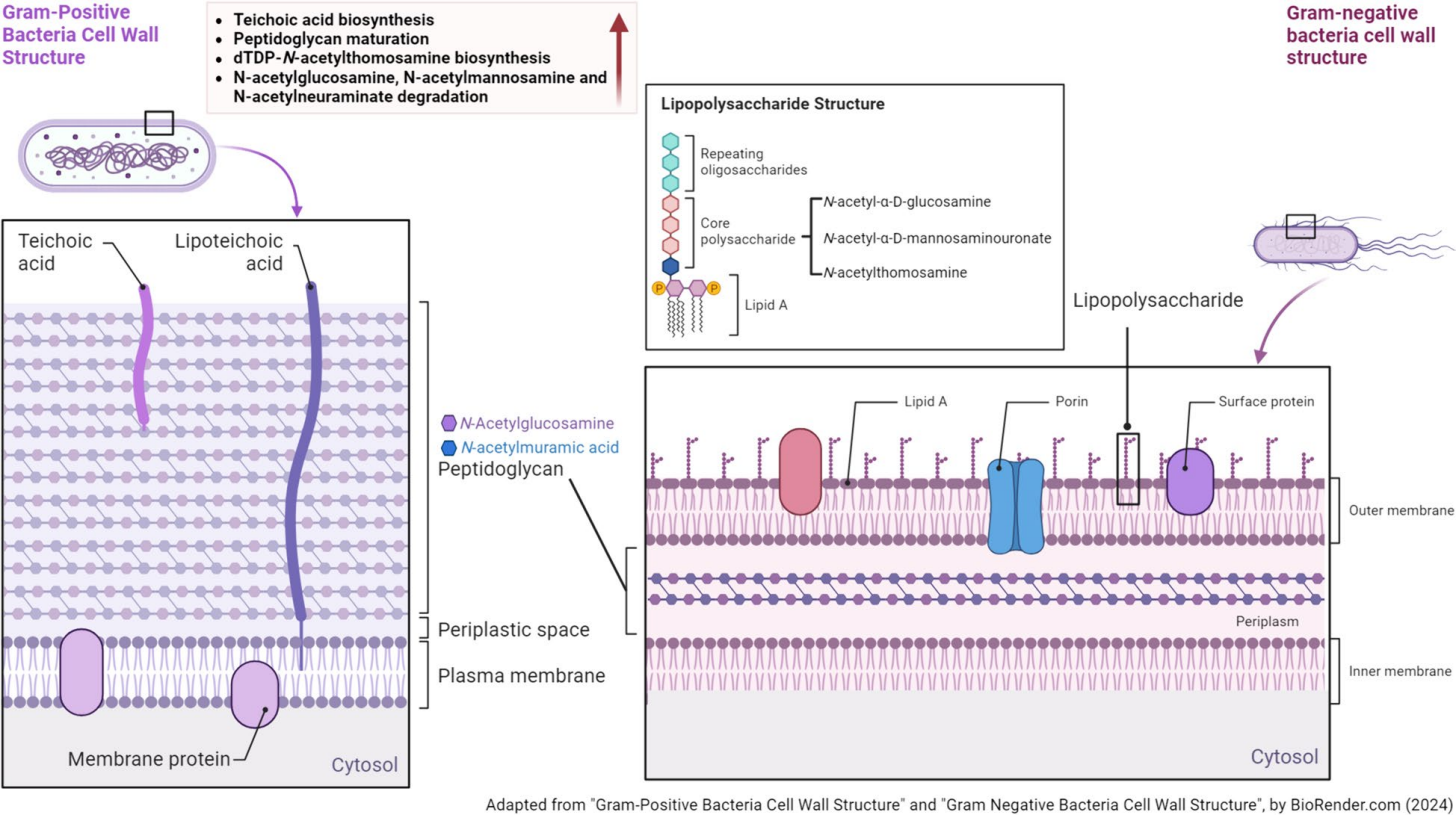
Predicted pathways related to bacterial wall structure synthesis altered by ambient organophosphorus pesticides exposure

We also predicted a higher abundance of several pathways related to the formation of bacterial walls with higher exposure to OP pesticides (Fig. 2). Teichoic acids are found in the cell wall of gram-positive bacteria and peptidoglycan is the main component in the envelope of both gram-positive and gram-negative bacteria. These two polymeric macromolecules are responsible for maintaining the structure, shape, and stability of bacteria, protecting the bacteria from environmental stress such as antimicrobial molecules, host interaction, and biofilm formation [59, 60]. They also play important roles in cell division and fundamental bacterial physiology [61]. In addition, increased biosynthesis and degradation of amino sugars (N-acetylglucosamine, N-acetylmannosamine and N-acetylneuraminate), and increased biosynthesis of nucleotides, nucleosides, and sugar nucleotides were predicted. These compounds are utilized in bacteria as precursors of DNA/RNA, and sources of carbon and energy. We suspect that these pathways reflect the collective increase in metabolic activity of the gut microbiota in defense against harmful changes in the gut environment such as chronic OP toxicity.

It is important to highlight that the altered predicted pathways we observed were found when we considered the gut microbiome as an ecosystem in its entirety. These changes do not indicate changes in any particular bacterial groups specifically.

In sensitivity analyses, the largest discrepancy in estimates of differential bacterial abundance was observed for a 0–5 year exposure window prior to fecal sample collection. As the actual window for ambient pesticide exposure to affect the microbiome is unknown, we believe this mainly results from sparser data for exposure: In the 0–5-year window, 19 participants previously considered highly exposed were moved into the low exposure group, leaving less participants in the exposed group (*N* = 17). In the analysis without PD status in the model, all results remained consistent in terms of direction of change. This indicates that PD status is not confounding the OP exposure related differences in the gut microbiome we observed.

The current understanding of associations between OPs and the gut microbiome is derived solely from animal models and in-vitro studies. OP treatment of animals resulted in changes of the host gastrointestinal tract such as shorter and thinner intestinal villi, decreased tight junction proteins, increased intestinal permeability and bacterial translocation [62–64]. Gut microbiome dysbiosis [63, 65], abundance changes of certain bacterial groups [9, 62, 63, 66–68], as well as altered metabolic function with OP exposure was also observed in animal models and human gut simulation systems [67, 69]. Manipulation of the gut microbiome (e.g., fecal microbiota transplants, administration of probiotics) in animal experiments alleviated OP toxicity, which underscores the essential role of the gut microbiome in the organismal response to OP exposure [53, 70–72]. However, findings from these experimental studies have been inconsistent and sometimes outright contradictory, possibly due to differences in animals used, specific pesticides or doses investigated or other features of the experimental design. Evidence from human studies of pesticide exposure and the gut microbiome is needed to elucidate real world exposure scenarios and vulnerabilities. To date, only a handful of human studies are available, as controlled human exposure trials are unethical and accurate exposure assessment in observational studies is challenging. A UK study investigating the fecal microbiome by estimating pesticide exposures from dietary questionnaire as well as measured pesticides metabolites in the urine of 65 twin pairs reported positive associations between OP metabolites (sum of dimethyl- and diethyl-containing metabolites) and several *Clostridium* spp [73]. A fecal microbiome study in Japan enrolled 38 healthy subjects, measured biomarkers of pesticides in urine and collected lifestyle information including diet; they only reported an increased abundance of *Agathobacter* as being associated with higher OP exposure [74]. These studies are small in size, and the pesticide biomarkers likely reflect only recent exposure, thus, they do not inform on microbiome changes from long-term exposure. Another study in the US found that the microbiome composition on indoor surfaces of farm worker homes were influenced by workers’ occupational exposure to OP pesticides, serving as an indicator for the influence that pesticide exposure has on the microbiome in general [75].

Our study is the largest microbiome study investigating the effect of OP pesticides in human samples to date. The California counties of Fresno, Tulare, and Kern are heavily agricultural with large-scale commercial applications of OP and other types of pesticides, and many have been shown to travel in the air to nearby areas and contaminate the outdoor and indoor environment of buildings [76, 77]. Thus, residents may inhale airborne pesticides or ingest them from contaminated soil, water, or food. Our PUR-based exposure assessment method has both strengths and limitations. This record-based system allows us to estimate long-term exposure at residential addresses without the potential for introducing recall bias. On the other hand, our measures of ambient pesticide exposure are probably affected by non-differential exposure misclassification as: 1) the actual exposure depends on where and how long a study participant spent time in contaminated areas, information that is not available; 2) we did not consider potential OP exposure from food residues or household pesticides use, as additional sources of OP exposures to the gut microbiome. Our current OP exposure estimate, therefore, may underestimate the actual OP exposure levels and their effect on the gut microbiome. Furthermore, the study sample had a mean age of 72 years and 61% had PD, limiting the generalizability of the study results to younger, healthier populations. In addition, as a number of factors can potentially affect the composition and abundance of gut bacteria over time, it is nevertheless generally believed that due to immunologic properties etc., the major gut bacteria composition within individuals are relatively robust and remain stable over time. In support of this, a study of 37 healthy adults collected fecal samples 2 to 13 times for up to 296 weeks apart and found that individual microbiota were fairly stable, with 60% of all strains remaining stable over the course of 5 years [78]. The National Institutes of Health Human Microbiome Project also reported on the strain stability of gut microbes over time [79]. Lastly, our sequencing and annotation pipeline had limited species-level resolution. Some of these limitations are expected to be resolved in the near future due to improvements in the size of the microbiome/metagenome reference database and the affordability of sequencing technology, such as full-length 16S or shotgun sequencing.

## Conclusion

In conclusion, our study provides evidence that exposure to OP is associated with changes in the abundance of several bacterial groups and differential functional capacity in metabolic pathways supported by the human gut microbiome. These findings support previous in-vivo and in-vitro studies and suggest that chronic and long-term OP pesticide exposures may also have an impact on the human gut microbiome. However, additional human studies are needed to confirm these results and elucidate possible health consequences of these observations.

### Supplementary Information

**Additional file 1: Table S1.** List of other pesticide groups. **Table S2.** Differential taxa abundance associated with organophosphorus pesticides - Main model and sensitivity analyses - Phylum (*N*=190). **Table S3.** Differential taxa abundance associated with organophosphorus pesticides - Main model and sensitivity analyses - Family (*N*=190). **Table S4.** Differential taxa abundance associated with organophosphorus pesticides - Main model and sensitivity analyses - Genus (*N*=190). **Table S5.** Differential taxa abundance associated with organophosphorus pesticides - Main model and sensitivity analyses - predicted Metacyc pathways (*N*=190). Supplementary Figures: **Figure S1.** Relative abundance plot at phylum level (Sorted by Firmicutes). **Figure S2.** Averaged relative taxa abundance grouped by organophosphorus pesticide exposures. **Figure S3.** Exposure windows of main model and sensitivity analyses. **Figure S4.** Distribution of organophosphorus pesticide exposures. **Figure S5.** Comparison of microbiome profile between organophosphorus exposure groups. **Figure S6.** Bacterial taxa associated with organophosphorus pesticide exposures. **Figure S7.** Comparison of predicted metagene diversity between organophosphorus pesticids exposure groups. **Figure S8.** Predicted Metacyc pathways associated with organophosphorus pesticide exposure, grouped by level 2 superclasses. **Figure S9.** Predicted Metacyc pathways associated with organophosphorus pesticides exposure, grouped by level 1 superclasses

## Data Availability

The data generated in this study are held by the study Principal Investigator Dr. Beate Ritz (britz@ucla.edu) and may be shared upon request for the purpose of replicating analyses results. The data are currently not deposited in publicly available repositories as data collection is ongoing. A comprehensive dataset will be made accessible in accordance with the requirements of funding agencies upon the completion of the study.

## Abbreviations

OP: Organophosphorus
GIS: Geographic information systems
PEG: Parkinson’s, Environment and Gene study
PD: Parkinson’s disease
ASV: Amplicon sequence variants
EC: Enzyme commission
PUR: Pesticide Use Reports
PERMANOVA: Permutation multivariate analysis of variance
SCFAs: Short-chain fatty acids
