# Supplementary material for: Ambient long-term exposure to organophosphorus pesticides and the human gut microbiome: an observational study
Source: Environ Health. 2024 Apr 16;23:41. doi: 10.1186/s12940-024-01078-y (PMC11020204; doi:10.1186/s12940-024-01078-y)

Supplementary Materials

Supplementary Tables:

Table S1. List of other Pesticides Groups

Table S2. Differential taxa abundance associated with organophosphorus pesticides - Main model and sensitivity analyses - Phylum (N=190).

Table S3. Differential taxa abundance associated with organophosphorus pesticides - Main model and sensitivity analyses - Family (N=190).

Table S4. Differential taxa abundance associated with organophosphorus pesticides - Main model and sensitivity analyses - Genus (N=190).

Table S5. Differential taxa abundance associated with organophosphorus pesticides - Main model and sensitivity analyses - predicted Metacyc pathways (N=190).

Supplementary Figures:

Figure S1. Relative abundance plot at phylum level (Sorted by Firmicutes)

Figure S2. Averaged relative taxa abundance grouped by organophosphorus pesticides exposure

Figure S3. Exposure windows of main model and sensitivity models

Figure S4. Distribution of organophosphorus pesticides exposure

Figure S5. Comparison of microbiome profile between organophosphorus exposure groups

Figure S6. Bacterial taxa associated with organophosphorus pesticides exposure

Figure S7. Comparison of predicted metagene diversity between organophosphorus pesticides exposure groups

Figure S8. Predicted Metacyc pathways associated with organophosphorus pesticides exposure, grouped by level 2 superclasses

Figure S9. Predicted Metacyc associated with organophosphorus pesticides exposure, grouped by level 1 superclasses

Table S1. List of other Pesticides Groups

**Fumigants**
CARBON DISULFIDE
CARBON TETRACHLORIDE
CHLOROPICRIN
DBCP
1,2-DICHLOROPROPANE, 1,3-DICHLOROPROPENE AND RELATED C3 COMPOUNDS
ETHYLENE DIBROMIDE
ETHYLENE OXIDE
METHYL BROMIDE
METHYL ISOTHIOCYANATE
OXYTHIOQUINOX
ALUMINUM PHOSPHIDE
1,3-DICHLOROPROPENE
METAM-SODIUM
POTASSIUM N-METHYLDITHIOCARBAMATE
SODIUM TETRATHIOCARBONATE
**Fungicides & Insecticides**
AMMONIA
COPPER CARBONATE, BASIC
COPPER NAPHTHENATE
COPPER SULFATE (PENTAHYDRATE)
COPPER OXIDE (OUS)
DINOCAP
LIME-SULFUR
OXYTHIOQUINOX
SILICA AEROGEL
SODIUM ARSENITE
SULFUR
DIAMMONIUM PHOSPHATE
PETROLEUM OIL, UNCLASSIFIED
SODIUM DODECYLBENZENE SULFONATE
CALCIUM CHLORIDE
SODIUM METASILICATE
SODIUM BENZENE SULFONATE
FREE FATTY ACIDS AND/OR AMINE SALTS
SODIUM ALKYLARYL SULFONATE
ALKYL BENZENE SULFONIC ACID
BENZOIC ACID
COCONUT DIETHANOLAMIDE
TRIFORINE
CINNAMALDEHYDE

ALPHA-ALKYL (SECONDARY C11-C15)-OMEGA-HYDROXYPOLY(OXYETHYLENE)
ISOPROPYLAMINE DODECYLBENZENE SULFONATE
OIL OF JOJOBA

Fungicides

DICLORAN
2-BUTOXYETHANOL
CALCIUM HYDROXIDE
CAPTAN
CITRIC ACID
COPPER HYDROXIDE
COPPER SALTS OF FATTY AND ROSIN ACIDS
COPPER OXYCHLORIDE
COPPER OXYCHLORIDE SULFATE
COPPER SULFATE (BASIC)
COPPER-ZINC SULFATE COMPLEX
DICHLONE
DICHLOROPHEN
MANCOZEB
ANILAZINE
FERBAM
CAPTAFOL
FOLPET
MANEB
NABAM
SULFURIC ACID
PCNB
ALUMINUM PHOSPHIDE
METIRAM
PROPIONIC ACID
PROPYLENE GLYCOL
PCP, SODIUM SALT
SODIUM BISULFITE
SODIUM DIMETHYL DITHIO CARBAMATE
TERRAZOLE
THIRAM
FENTIN HYDROXIDE
METAM-SODIUM
ZINEB
ZIRAM
2,4-XYLENOL
UREA
CHLOROTHALONIL
COPPER

COPPER DIHYDRAZINIUM SULFATE
2-METHYL-1-NAPHTHALENE ACETAMIDE
SODIUM CARBONATE
PHOSPHORIC ACID
SODIUM BISULFATE
ACETIC ACID
POTASSIUM N-METHYLDITHIOCARBAMATE
LIME
POTASSIUM SORBATE
POTASSIUM HYDROXIDE
OXYCARBOXIN
BENOMYL
TRISODIUM PHOSPHATE
DIDECYL DIMETHYL AMMONIUM CHLORIDE
THIOPHANATE
THIOPHANATE-METHYL
OCTYL DECYL DIMETHYL AMMONIUM CHLORIDE
DIOCTYL DIMETHYL AMMONIUM CHLORIDE
COPPER-ZINC SULFATE COMPLEX, MONOHYDRATE
CARBOXIN
COPPER SULFATE, MONOHYDRATE
OXYTETRACYCLINE HYDROCHLORIDE
ALKYL (68%C12, 32%C14) DIMETHYLETHYLBENZYL AMMONIUM CHLORIDE
POTASSIUM PEROXYMONOSULFATE
TETRAPOTASSIUM PYROPHOSPHATE
FENARIMOL
AGROBACTERIUM RADIOBACTER
1-BROMO-3-CHLORO-5,5-DIMETHYL HYDANTOIN
IPRODIONE
DODEMORPH ACETATE
VINCLOZOLIN
METALAXYL
TRIADIMEFON
FOSETYL-AL
MYCLOBUTANIL
TRIFLUMIZOLE
SODIUM TETRATHIOCARBONATE
PROPICONAZOLE
AMPELOMYCES QUISQUALIS
NONANOIC ACID
PSEUDOMONAS FLUORESCENS, STRAIN A506
COPPER AMMONIUM COMPLEX
OXYTETRACYCLINE, CALCIUM COMPLEX

STREPTOMYCIN SULFATE
TEBUCONAZOLE
FENBUCONAZOLE
TRICHODERMA HARZIANUM RIFAI STRAIN KRL-AG2
CYPRODINIL
CYMOXANIL
DIMETHOMORPH
MEFENOXAM
PROPAMOCARB HYDROCHLORIDE
AZOXYSTROBIN
POTASSIUM BICARBONATE

Insecticides

ARAMITE
ARSENIC ACID
BACILLUS THURINGIENSIS (BERLINER)
CARBARYL
CARBOFURAN
FORMETANATE HYDROCHLORIDE
CHLORDANE
CHLOROBENZILATE
BUTOXY POLYPROPYLENE GLYCOL
CRYOLITE
DIATOMACEOUS EARTH
DIELDRIN
ENDOSULFAN
SODIUM ARSENATE
CHLORDIMEFORM
CHLORDIMEFORM HYDROCHLORIDE
DICOFOL
LEAD ARSENATE (STANDARD)
LEAD ARSENATE, BASIC
2-(2-BUTOXY ETHOXY) ETHYL THIOCYANATE
LINDANE
METHIOCARB
METHOMYL
METHOXYCHLOR
METHYL BROMIDE
METHYL ISOTHIOCYANATE
MINERAL OIL
PROPARGITE
DIENOCHLOR
CAPSICUM OLEORESIN
PETROLEUM HYDROCARBONS

PINE OIL
PYRETHRINS
ROTENONE
RYANODINE ALKALOID
SABADILLA ALKALOIDS
ALDICARB
TETRADIFON
TOXAPHENE
1080
PETROLEUM DISTILLATES
BORIC ACID
XYLENE RANGE AROMATIC SOLVENT
DODECYLBENZENE SULFONIC ACID
SODIUM DIISOOCTYLSULFOSUCCINATE
LIMONENE
AMMONIUM TALL OIL FATTY ACID SOAP
TRIETHANOLAMINE
COTTONSEED OIL
AMMONIUM OLEATE
VEGETABLE OIL
POTASH SOAP
CYHEXATIN
SULCOFURON-SODIUM
PETROLEUM DISTILLATES, AROMATIC
DIMETHYLPOLYSILOXANE
PIRIMICARB
FENBUTATIN-OXIDE
OXAMYL
BENDIOCARB
FENVALERATE
TRIETHANOLAMINE SULFONATE
DIFLUBENZURON
PERMETHRIN
AMITRAZ
PETROLEUM OIL, PARAFFIN BASED
SODIUM DIOCTYLSULFOSUCCINATE
KEROSENE
PHENOTHRIN
PETROLEUM DISTILLATES, REFINED
PETROLEUM NAPHTHENIC OILS
RESMETHRIN
FLUCYTHRINATE
CYPERMETHRIN

TAU-FLUVALINATE
THIODICARB
GARLIC
CYFLUTHRIN
FENPROPATHRIN
CLOFENTEZINE
ABAMECTIN
CYROMAZINE
LAMBDA-CYHALOTHRIN
BIFENTHRIN
ESFENVALERATE
AZADIRACHTIN
TRALOMETHRIN
SOYBEAN OIL
ENCAPSULATED DELTA ENDOTOXIN OF BACILLUS THURINGIENSIS VAR. KURSTAKI IN KILLED PSEUDOMONAS FLUORESCENS
BACILLUS THURINGIENSIS (BERLINER), SUBSP. AIZAWAI, GC-91 PROTEIN
IMIDACLOPRID
BACILLUS THURINGIENSIS (BERLINER), SUBSP. AIZAWAI, SEROTYPE H-7
BACILLUS THURINGIENSIS (BERLINER), SUBSP. ISRAELENSIS, SEROTYPE H-14
BACILLUS THURINGIENSIS (BERLINER), SUBSP. KURSTAKI, SEROTYPE 3A,3B
BACILLUS THURINGIENSIS (BERLINER), SUBSP. KURSTAKI, STRAIN EG 2348
BACILLUS THURINGIENSIS (BERLINER), SUBSP. KURSTAKI, STRAIN EG2371
BACILLUS THURINGIENSIS (BERLINER), SUBSP. KURSTAKI, STRAIN SA-11
(S)-CYPERMETHRIN
CHLORFENAPYR
TEBUFENOZIDE
PYRIDABEN
CLARIFIED HYDROPHOBIC EXTRACT OF NEEM OIL
SPINOSAD
BACILLUS THURINGIENSIS SUBSPECIES KURSTAKI, GENETICALLY ENGINEERED STRAIN EG7841 LEPIDOPTERAN ACTIVE TOXIN
BEAUVERIA BASSIANA STRAIN GHA
BETA-PINENE POLYMER
PYRIPROXYFEN
BACILLUS THURINGIENSIS, SUBSP. KURSTAKI, STRAIN HD-1
EMULSIFIABLE METHYLATED VEGETABLE OIL

Herbicides

AMITROLE
CACODYLIC ACID
MSMA
ARSENIC ACID
ATRAZINE

TRIALLATE
BENEFIN
BARBAN
BROMACIL
CALCIUM CYANAMIDE
CALCIUM HYDROXIDE
DICHLOBENIL
ALLIDOCHLOR
CHLORPROPHAM
FLUOMETURON
CHLORTHAL-DIMETHYL
DICAMBA
DICHLOROPHEN
DIPHENAMID
DIQUAT DIBROMIDE
DIURON
DINOSEB, TRIETHANOLAMINE SALT
DINOSEB
DINOSEB, AMINE SALT
DINOSEB, AMMONIUM SALT
DSMA
ENDOTHALL, DISODIUM SALT
EPTC
SODIUM ARSENATE
FERROUS SULFATE
PROPHAM
SILVEX, PROPYLENE GLYCOL BUTYL ETHER ESTER
LEAD ARSENATE (STANDARD)
LEAD ARSENATE, BASIC
LINURON
MCPP
METHYL BROMIDE
METHYL ISOTHIOCYANATE
MONURON
NABAM
NAPTALAM, SODIUM SALT
SULFURIC ACID
MOLINATE
PARAQUAT BIS(METHYLSULFATE)
NITRALIN
PROMETRYN
PROPANIL
PROPAZINE

PYRAZON
PROPACHLOR
CYCLOATE
SIMAZINE
TERBACIL
SODIUM ARSENITE
SODIUM CHLORATE
TRICHLOROACETIC ACID, SODIUM SALT
BUTYLATE
CHLOROXURON
PEBULATE
NITROFEN
PICLORAM
TRIFLURALIN
FENTIN HYDROXIDE
METAM-SODIUM
2,4-D
DIAMMONIUM PHOSPHATE
ZINC SULFATE
PHENMEDIPHAM
ALACHLOR
KARBUTILATE
PROPYZAMIDE
PETROLEUM OIL, UNCLASSIFIED
MCPA, BUTOXYETHANOL ESTER
MCPA, DIMETHYLAMINE SALT
MCPA, ISOOCTYL ESTER
MCPA, SODIUM SALT
2,4-D, ALKANOLAMINE SALTS (ETHANOL AND ISOPROPANOL AMINES)
2,4-D, BUTOXYETHANOL ESTER
2,4-D, BUTOXYPROPYL ESTER
2,4-D, BUTYL ESTER
2,4-D, DIETHANOLAMINE SALT
2,4-D, DIMETHYLAMINE SALT
2,4-D, ISOOCTYL ESTER
2,4-D, ISOPROPYL ESTER
2,4,5-T, BUTOXYETHANOL ESTER
SILVEX, BUTOXYETHANOL ESTER
BROMOXYNIL OCTANOATE
4-(2,4-DB), BUTOXYETHANOL ESTER
4-(2,4-DB), DIMETHYLAMINE SALT
DICAMBA, DIMETHYLAMINE SALT
SODIUM CARBONATE

PHOSPHORIC ACID
SODIUM BISULFATE
CALCIUM CHLORIDE
DICHLORPROP, BUTOXYETHANOL ESTER
ACETIC ACID
LIME
MCPP, DIMETHYLAMINE SALT
DALAPON, SODIUM SALT
2,4-D, N-OLEYL-1,3-PROPYLENEDIAMINE SALT
2,4-D, TRIETHYLAMINE SALT
FREE FATTY ACIDS AND/OR AMINE SALTS
2,4-D, PROPYL ESTER
MCPP, POTASSIUM SALT
POTASSIUM HYDROXIDE
ENDOTHALL, MONO (N,N-DIETHYL ALKYLAMINE) SALT
ENDOTHALL, DI (N,N-DIETHYLALKYLAMINE) SALT
ENDOTHALL, DIPOTASSIUM SALT
AMMONIUM SULFATE
4-(2,4-DB), ISOOCTYL ESTER
TRISODIUM PHOSPHATE
POTASH SOAP
PARAQUAT DICHLORIDE
2,4-D, 2-ETHYLHEXYL ESTER
CYANAZINE
SODIUM CACODYLATE
DINITRAMINE
TERBUTRYN
METRIBUZIN
NAPROPAMIDE
DALAPON, MAGNESIUM SALT
DESMEDIPHAM
BUTRALIN
TEBUTHIURON
FERRIC SULFATE (ANHYDROUS)
PETROLEUM DISTILLATES, AROMATIC
FLUCHLORALIN
GLYPHOSATE, ISOPROPYLAMINE SALT
POTASSIUM PEROXYMONOSULFATE
ORYZALIN
HEXAZINONE
PROFLURALIN
ETHOFUMESATE
TETRAPOTASSIUM PYROPHOSPHATE

FOSAMINE, AMMONIUM SALT
PENDIMETHALIN
DIFENZOQUAT METHYL SULFATE
THIOBENCARB
BENTAZON, SODIUM SALT
OXYFLUORFEN
VERNOLATE
DIETHATYL-ETHYL
METOLACHLOR
OXADIAZON
NORFLURAZON
DICLOFOP-METHYL
ENDOTHALL, MONO [N,N-DIMETHYL ALKYLAMINE] SALT
1-BROMO-3-CHLORO-5,5-DIMETHYL HYDANTOIN
MALEIC HYDRAZIDE, POTASSIUM SALT
TRICLOPYR, TRIETHYLAMINE SALT
CHLORSULFURON
BROMOXYNIL BUTYRATE
ETHALFLURALIN
TRICLOPYR, BUTOXYETHYL ESTER
SETHOXYDIM
FLUAZIFOP-BUTYL
PRODIAMINE
HYDROGEN CYANAMIDE
2-(2,4-DP), DIMETHYLAMINE SALT
BENSULFURON METHYL
UREA DIHYDROGEN SULFATE
ISOXABEN
GLYPHOSATE, MONOAMMONIUM SALT
DITHIOPYR
ZINC
FENOXAPROP-ETHYL
CORN PRODUCT, HYDROLYZED
GLYPHOSATE-TRIMESIUM
IMAZETHAPYR
IMAZETHAPYR, AMMONIUM SALT
TRINEXAPAC-ETHYL
NONANOIC ACID
GLYPHOSATE
CLETHODIM
NICOSULFURON
RIMSULFURON
TRIFLUSULFURON-METHYL

HALOSULFURON-METHYL
PYRIDATE
PYRITHIOBAC-SODIUM
THIAZOPYR
DIGLYCOLAMINE SALT OF 3,6-DICHLORO-O-ANISIC ACID
BROMOXYNIL HEPTANOATE
CLOPYRALID, MONOETHANOLAMINE SALT

Table S2. Differential taxa abundance associated with organophosphorus pesticides - Main model and sensitivity analyses - Phylum (N=190).

		**Main Model[a] (10 year exposure window)**			**Sensitivity Analyses 1[b] (5 year exposure window - 5 year lag)**			**Sensitivity Analyses 2[c] (5 year exposure window)**			**Sensitivity Analyses 3[d] (Excluded PD variable from main model)**		
**Kingdom**	**Phylum**	**Log2 FC**	**SE**	**Adj P**	**Log2 FC**	**SE**	**Adj P**	**Log2 FC**	**SE**	**Adj P**	**Log2 FC**	**SE**	**Adj P**
Bacteria	Synergistetes	-1.79	0.92	2.478E-01	-1.68	0.96	2.790E-01	-2.20	1.21	2.040E-01	-2.07	0.93	1.198E-01
Bacteria	Actinobacteria	0.57	0.31	2.478E-01	0.67	0.32	1.685E-01	0.79	0.40	1.742E-01	0.52	0.31	3.091E-01
Bacteria	Firmicutes	-0.15	0.13	4.588E-01	-0.18	0.13	4.002E-01	0.20	0.17	4.123E-01	-0.15	0.13	4.330E-01
Bacteria	Proteobacteria	-0.31	0.27	4.590E-01	-0.37	0.28	4.145E-01	-0.06	0.36	9.401E-01	-0.31	0.27	4.330E-01
Bacteria	Bacteroidetes	-0.08	0.17	8.532E-01	-0.06	0.18	8.560E-01	-0.27	0.22	4.123E-01	-0.08	0.17	7.971E-01
Bacteria	Verrucomicrobia	0.19	0.35	8.532E-01	0.19	0.37	8.196E-01	-0.95	0.46	1.742E-01	0.17	0.36	7.971E-01
Archaea	Euryarchaeota	-0.06	0.61	9.617E-01	-0.11	0.63	8.957E-01	-1.33	0.79	2.040E-01	0.07	0.61	9.438E-01

[a] 10-year exposure window adjusted for sex, race, age, Parkinson's disease status, pesticides co-exposure, and sequencing platform.

[b] 6-to-10 years exposure window, aadjusted for sex, race, age, Parkinson's disease status, pesticides co-exposure, and sequencing platform.

[c] 0-to-5 years exposure window, aadjusted for sex, race, age, Parkinson's disease status, pesticides co-exposure, and sequencing platform.

[d] 10-year exposure window, aadjusted for sex, race, age, pesticides co-exposure, and sequencing platform.

Abbreviations: Log2FC: Log2 Fold Change; SE: Standard Error: Adj: Adjusted.

Table S3. Differential taxa abundance associated with organophosphorus pesticides - Main model and sensitivity analyses - Family (N=190).

		Main Model[a] (10 year exposure window)			Sensitivity Analyses 1[b] (5 year exposure window - 5 year lag)			Sensitivity Analyses 2[c] (5 year exposure window)			Sensitivity Analyses 3[d] (Excluded PD variable from main model)		
**Order**	**Family**	**Log2 FC**	**SE**	**Adj P**	**Log2 FC**	**SE**	**Adj P**	**Log2 FC**	**SE**	**Adj P**	**Log2 FC**	**SE**	**Adj P**
Bacteroidales	Barnesiellaceae	0.59	0.05	8.739E-35	0.43	0.05	4.491E-18	1.37	1.15	4.615E-01	0.58	0.05	5.142E-35
Coriobacteriales	Coriobacteriales_Incertae_Sedis	-1.02	0.08	4.644E-35	-1.25	0.80	3.168E-01	0.01	0.12	9.649E-01	-1.04	0.08	3.152E-36
Synergistales	Synergistaceae	-2.41	0.93	6.027E-02	-2.36	0.97	7.924E-02	-2.44	1.23	1.576E-01	-2.38	0.94	6.789E-02
Clostridiales	Clostridiaceae_1	-1.09	0.48	1.100E-01	-1.51	0.50	2.368E-02	-0.53	0.64	6.138E-01	-1.09	0.49	1.164E-01
Clostridiales	Lachnospiraceae	0.31	0.14	1.216E-01	0.32	0.15	1.174E-01	0.76	0.18	2.083E-04	0.29	0.14	1.658E-01
Lactobacillales	Enterococcaceae	-2.00	0.97	1.599E-01	-2.98	1.00	2.539E-02	-2.06	1.29	2.864E-01	-2.08	0.98	1.476E-01
Clostridiales	Clostridiales_vadinBB60_group	0.13	0.06	1.900E-01	0.14	0.07	1.329E-01	-1.21	0.11	5.763E-28	0.10	0.06	3.719E-01
Betaproteobacteriales	Burkholderiaceae	-0.73	0.38	1.942E-01	-0.85	0.39	1.268E-01	-0.35	0.50	7.045E-01	-0.66	0.38	2.543E-01
Coriobacteriales	Eggerthellaceae	0.65	0.34	1.962E-01	0.61	0.35	2.658E-01	0.65	0.44	3.284E-01	0.66	0.34	1.938E-01
Clostridiales	Unspecified	-1.00	0.59	2.617E-01	-0.99	0.61	3.111E-01	-2.07	0.76	3.480E-02	-0.90	0.60	3.616E-01
Bacteroidales	Rikenellaceae	-0.47	0.28	2.617E-01	-0.41	0.29	3.559E-01	-0.67	0.36	1.895E-01	-0.41	0.28	3.728E-01
Bacteroidales	Marinifilaceae	-0.62	0.42	3.395E-01	-0.60	0.44	4.017E-01	-0.52	0.04	5.790E-36	-0.56	0.42	4.593E-01
Clostridiales	Eubacteriaceae	0.91	0.66	3.865E-01	1.04	0.68	3.184E-01	-0.27	0.87	8.746E-01	0.77	0.70	5.399E-01
Clostridiales	Peptostreptococcaceae	-0.47	0.35	4.020E-01	-0.61	0.37	2.971E-01	-0.35	0.46	6.804E-01	-0.31	0.36	6.429E-01
Clostridiales	Family_XI	-0.89	0.83	5.101E-01	-0.91	0.86	5.216E-01	-3.17	1.15	3.298E-02	-0.57	0.84	7.414E-01
Clostridiales	Christensenellaceae	-0.46	0.42	5.101E-01	-0.64	0.44	3.525E-01	-1.17	0.55	1.200E-01	-0.46	0.42	5.399E-01
Enterobacteriales	Enterobacteriaceae	-0.41	0.41	5.456E-01	-0.48	0.43	5.131E-01	-0.19	0.54	8.560E-01	-0.42	0.41	5.733E-01
Pasteurellales	Pasteurellaceae	-0.57	0.60	5.655E-01	-0.75	0.61	4.742E-01	-1.04	0.80	4.154E-01	-0.86	0.61	3.987E-01
Clostridiales	Ruminococcaceae	-0.12	0.13	5.863E-01	-0.13	0.14	5.724E-01	-0.01	0.18	9.649E-01	-0.13	0.13	6.220E-01
Bifidobacteriales	Bifidobacteriaceae	0.40	0.47	6.178E-01	0.46	0.49	5.673E-01	0.70	0.62	4.771E-01	0.35	0.47	7.052E-01
Desulfovibrionales	Desulfovibrionaceae	-0.28	0.35	6.355E-01	-0.40	0.37	5.216E-01	-0.17	0.46	8.560E-01	-0.27	0.35	7.052E-01
Methanobacteriales	Methanobacteriaceae	-0.49	0.62	6.355E-01	-0.56	0.64	5.912E-01	-1.43	0.80	2.158E-01	-0.30	0.62	8.180E-01
Bacteroidales	Tannerellaceae	-0.22	0.27	6.355E-01	-0.19	0.28	6.936E-01	-0.09	0.36	9.041E-01	-0.20	0.27	7.052E-01
Verrucomicrobiales	Akkermansiaceae	0.28	0.35	6.355E-01	0.30	0.36	6.177E-01	-0.51	0.46	4.779E-01	0.29	0.35	6.674E-01
Selenomonadales	Veillonellaceae	-0.20	0.48	8.257E-01	-0.19	0.49	8.325E-01	0.43	0.63	7.199E-01	-0.14	0.48	8.900E-01
Erysipelotrichales	Erysipelotrichaceae	0.08	0.21	8.365E-01	0.11	0.22	7.838E-01	0.27	0.27	5.485E-01	0.08	0.21	8.643E-01

Bacteroidales	Prevotellaceae	0.18	0.51	8.500E-01	-0.53	0.53	5.422E-01	0.25	0.67	8.560E-01	0.16	0.51	8.891E-01
Bacteroidales	Bacteroidaceae	0.05	0.18	8.779E-01	0.14	0.19	6.468E-01	-0.03	0.23	9.529E-01	0.03	0.18	9.362E-01
Actinomycetales	Actinomycetaceae	-0.18	0.59	8.779E-01	-0.59	0.61	5.648E-01	0.05	0.77	9.696E-01	-0.20	0.59	8.859E-01
Clostridiales	Family_XIII	0.10	0.40	8.998E-01	0.14	0.41	8.467E-01	-0.14	0.53	8.994E-01	0.13	0.40	8.859E-01
Selenomonadales	Acidaminococcaceae	-0.04	0.38	9.640E-01	-0.04	0.40	9.558E-01	-0.16	0.50	8.746E-01	-0.04	0.38	9.497E-01
Lactobacillales	Lactobacillaceae	-0.04	0.47	9.640E-01	-0.22	0.49	7.995E-01	0.12	0.62	9.236E-01	0.16	0.48	8.859E-01
Lactobacillales	Streptococcaceae	-0.02	0.29	9.658E-01	-0.35	0.30	4.849E-01	0.71	0.37	1.864E-01	-0.04	0.29	9.497E-01

[a] 10-year exposure window adjusted for sex, race, age, Parkinson's disease status, pesticides co-exposure, and sequencing platform.

[b] 6-to-10 years exposure window, aadjusted for sex, race, age, Parkinson's disease status, pesticides co-exposure, and sequencing platform.

[c] 0-to-5 years exposure window, aadjusted for sex, race, age, Parkinson's disease status, pesticides co-exposure, and sequencing platform.

[d] 10-year exposure window, aadjusted for sex, race, age, pesticides co-exposure, and sequencing platform.

Abbreviations: Log2FC: Log2 Fold Change; SE: Standard Error: Adj: Adjusted.

Table S4. Differential taxa abundance associated with organophosphorus pesticides - Main model and sensitivity analyses - Genus (N=190).

		**Main Model[a] (10 year exposure window)**			**Sensitivity Analyses 1[b] (5 year exposure window - 5 year lag)**			**Sensitivity Analyses 2[c] (5 year exposure window)**			**Sensitivity Analyses 3[d] (Excluded PD variable from main model)**		
**Family**	**Genus**	**Log2 FC**	**SE**	**Adj P**	**Log2 FC**	**SE**	**Adj P**	**Log2 FC**	**SE**	**Adj P**	**Log2 FC**	**SE**	**Adj P**
Lachnospiraceae	*Sellimonas*	0.94	0.04	1.91E-113	0.94	0.77	4.51E-01	0.95	0.04	4.06E-109	0.73	0.76	6.17E-01
Burkholderiaceae	*Sutterella*	0.59	0.03	1.62E-69	0.33	0.03	4.31E-22	0.21	0.04	1.48E-06	0.57	0.03	4.04E-66
Lachnospiraceae	*Blautia*	0.53	0.17	6.61E-03	0.55	0.17	7.25E-03	0.87	0.21	2.79E-04	0.51	0.17	1.03E-02
Ruminococcaceae	*Ruminococcaceae_UCG-014*	0.40	0.04	3.84E-19	0.36	0.05	1.86E-14	-0.58	0.07	8.21E-16	0.39	0.04	4.66E-18
Lachnospiraceae	*UC5-1-2E3*	0.38	0.05	6.20E-14	0.49	0.05	4.31E-20	-0.28	0.06	2.66E-05	0.36	0.05	1.21E-12
Ruminococcaceae	*Ruminococcaceae_UCG-010*	0.34	0.06	7.81E-07	0.20	0.07	9.26E-03	-0.86	0.10	9.69E-16	0.36	0.06	1.20E-07
Lachnospiraceae	*Lachnospiraceae_UCG-004*	0.32	0.05	4.07E-10	0.45	0.05	8.77E-17	-0.04	0.06	7.40E-01	0.26	0.05	3.58E-07
Lachnospiraceae	*Tyzzerella_4*	0.31	0.04	1.17E-16	0.20	0.04	2.26E-07	-0.07	0.04	2.47E-01	0.30	0.04	9.97E-16
Clostridiales_vadinBB60_group	Unspecified	0.28	0.06	2.47E-06	0.28	0.06	4.02E-06	-1.61	0.11	1.42E-48	0.27	0.06	7.32E-06
Lachnospiraceae	*Coprococcus_1*	0.23	0.02	4.85E-20	0.07	0.03	2.60E-02	0.20	0.03	2.22E-08	0.24	0.02	1.34E-20
Lachnospiraceae	*CAG-56*	0.20	0.04	3.48E-07	0.07	0.04	2.62E-01	-0.15	0.05	1.49E-02	0.19	0.04	9.77E-07
Ruminococcaceae	*Ruminococcaceae_UCG-004*	0.11	0.03	7.23E-04	0.16	0.03	1.46E-07	-0.65	0.04	4.51E-50	0.11	0.03	3.52E-04
Acidaminococcaceae	*Acidaminococcus*	0.07	0.02	4.83E-03	0.03	0.02	3.50E-01	-0.04	1.40	9.89E-01	0.08	0.02	2.70E-03
Lachnospiraceae	*Lachnospiraceae_FCS020_group*	-0.15	0.06	3.94E-02	-0.06	0.06	5.48E-01	0.30	0.08	5.26E-04	-0.18	0.06	5.82E-03
Veillonellaceae	*Dialister*	-0.17	0.02	1.38E-20	-0.13	0.02	3.26E-11	0.50	0.02	2.28E-106	-0.19	0.02	8.74E-25
Lachnospiraceae	*Tyzzerella*	-0.20	0.06	4.04E-03	-0.07	0.06	5.14E-01	1.39	0.10	8.30E-43	-0.26	0.06	9.46E-05
Ruminococcaceae	*DTU089*	-0.35	0.06	3.87E-09	-0.42	0.06	2.71E-12	-0.88	0.09	7.60E-22	-0.33	0.06	3.70E-08
Erysipelotrichaceae	*Holdemania*	-0.43	0.12	1.09E-03	-0.46	0.12	8.00E-04	0.34	0.16	8.49E-02	-0.43	0.12	1.24E-03
Ruminococcaceae	*Anaerotruncus*	-0.63	0.11	8.87E-08	-0.54	0.11	1.25E-05	-1.67	0.23	1.13E-12	-0.57	0.11	1.21E-06
Erysipelotrichaceae	*Turicibacter*	-1.54	0.61	4.35E-02	-1.49	0.63	6.85E-02	-0.81	0.81	5.50E-01	-1.18	0.62	1.69E-01
Burkholderiaceae	*Parasutterella*	-2.13	0.49	6.25E-05	-2.12	0.51	1.45E-04	-1.01	0.65	2.89E-01	-2.07	0.49	1.11E-04
Synergistaceae	*Cloacibacillus*	-2.47	0.93	3.18E-02	-2.44	0.97	4.52E-02	-2.93	1.24	6.23E-02	-2.64	0.95	2.25E-02
Lachnospiraceae	*Lachnospiraceae_UCG-001*	0.09	0.04	6.16E-02	-0.04	0.04	5.78E-01	-0.11	0.05	1.08E-01	0.07	0.04	1.58E-01
Ruminococcaceae	*Butyricicoccus*	-0.07	0.03	6.90E-02	-0.24	0.03	1.89E-15	0.05	0.61	9.76E-01	-0.08	0.03	1.42E-02
Clostridiaceae_1	*Clostridium_sensu_stricto_1*	-1.12	0.50	8.80E-02	-1.58	0.52	9.87E-03	-0.73	0.66	4.96E-01	-1.14	0.50	8.36E-02
Enterococcaceae	*Enterococcus*	-2.14	0.98	9.44E-02	-3.11	1.01	8.94E-03	-2.37	1.32	1.99E-01	-2.25	0.99	8.51E-02
Marinifilaceae	*Odoribacter*	-0.94	0.46	1.21E-01	-1.01	0.47	1.05E-01	-0.97	0.60	2.65E-01	-0.89	0.46	1.61E-01

Rikenellaceae	*Alistipes*	-0.57	0.29	1.44E-01	-0.58	0.30	1.60E-01	-0.92	0.37	5.10E-02	-0.49	0.29	2.46E-01
Peptostreptococcaceae	*Romboutsia*	-0.85	0.47	1.92E-01	-0.92	0.48	1.67E-01	-0.84	0.61	3.64E-01	-0.55	0.47	4.99E-01
Tannerellaceae	*Parabacteroides*	-0.48	0.27	2.06E-01	-0.49	0.28	2.12E-01	-0.30	0.35	6.31E-01	-0.45	0.27	2.64E-01
Ruminococcaceae	*Ruminococcaceae_UCG-013*	0.68	0.38	2.06E-01	0.52	0.40	4.16E-01	1.30	0.50	3.54E-02	0.56	0.39	3.61E-01
Erysipelotrichaceae	*Erysipelatoclostridium*	0.77	0.45	2.28E-01	0.83	0.47	2.05E-01	1.46	0.58	4.57E-02	0.88	0.45	1.60E-01
Veillonellaceae	*Veillonella*	-0.85	0.51	2.44E-01	-0.99	0.53	1.74E-01	-0.81	0.68	4.54E-01	-0.93	0.51	1.98E-01
Lachnospiraceae	*Lachnoclostridium*	0.27	0.17	3.00E-01	0.33	0.18	1.84E-01	0.52	0.23	7.65E-02	0.27	0.17	3.17E-01
Pasteurellaceae	*Haemophilus*	-0.82	0.59	3.75E-01	-0.95	0.61	2.94E-01	-0.95	0.80	4.55E-01	-1.13	0.60	1.80E-01
Unspecified	Unspecified Clostridiales	-0.81	0.59	3.85E-01	-0.83	0.61	3.89E-01	-2.24	0.76	1.41E-02	-0.65	0.60	5.39E-01
Bifidobacteriaceae	*Bifidobacterium*	0.64	0.48	3.91E-01	0.68	0.49	3.82E-01	0.75	0.63	4.44E-01	0.61	0.48	4.46E-01
Lachnospiraceae	*Dorea*	0.41	0.31	4.00E-01	0.42	0.32	4.03E-01	0.69	0.40	2.31E-01	0.42	0.31	4.11E-01
Erysipelotrichaceae	*Faecalitalea*	-1.05	0.79	4.01E-01	-1.06	0.82	4.12E-01	1.16	1.01	4.72E-01	-1.18	0.79	3.29E-01
Ruminococcaceae	*Ruminococcus_2*	-0.55	0.42	4.10E-01	-0.63	0.44	3.50E-01	-1.13	0.55	1.19E-01	-0.54	0.42	4.46E-01
Ruminococcaceae	*Ruminococcaceae_UCG-002*	-0.52	0.41	4.26E-01	-0.56	0.42	4.00E-01	-1.56	0.52	1.32E-02	-0.49	0.41	4.70E-01
Enterobacteriaceae	*Escherichia/Shigella*	-0.55	0.43	4.27E-01	-0.60	0.45	3.96E-01	-0.22	0.57	8.49E-01	-0.57	0.44	4.29E-01
Streptococcaceae	*Lactococcus*	0.89	0.73	4.59E-01	0.81	0.76	5.26E-01	2.74	0.93	1.37E-02	1.14	0.73	3.01E-01
Methanobacteriaceae	*Methanobrevibacter*	-0.77	0.64	4.66E-01	-0.88	0.67	4.02E-01	-1.74	0.83	1.09E-01	-0.43	0.65	7.66E-01
Desulfovibrionaceae	*Bilophila*	-0.50	0.45	5.09E-01	-0.49	0.46	5.26E-01	-0.26	0.59	8.36E-01	-0.48	0.45	5.48E-01
Ruminococcaceae	*Ruminiclostridium_5*	-0.26	0.25	5.36E-01	-0.24	0.26	5.86E-01	-0.42	0.32	3.95E-01	-0.27	0.25	5.44E-01
Erysipelotrichaceae	Unspecified	0.55	0.53	5.49E-01	0.55	0.55	5.48E-01	0.39	0.69	7.62E-01	0.61	0.53	4.99E-01
Lachnospiraceae	*Lachnospira*	-0.34	0.33	5.49E-01	-0.23	0.34	7.27E-01	-1.05	0.43	5.03E-02	-0.37	0.33	5.12E-01
Peptostreptococcaceae	*Terrisporobacter*	-0.98	0.96	5.63E-01	-1.22	1.00	4.51E-01	-1.43	1.26	4.76E-01	-0.76	0.97	7.17E-01
Ruminococcaceae	*Ruminococcaceae_UCG-003*	-0.03	0.03	5.63E-01	-0.03	0.03	6.51E-01	-0.90	0.05	2.16E-63	-0.04	0.03	4.88E-01
Erysipelotrichaceae	*Erysipelotrichaceae_UCG-003*	0.65	0.64	5.63E-01	0.79	0.66	4.63E-01	-0.41	0.84	8.08E-01	0.63	0.64	6.15E-01
Lachnospiraceae	*Agathobacter*	-0.29	0.30	5.76E-01	-0.20	0.31	7.36E-01	0.23	0.39	7.54E-01	-0.29	0.30	6.15E-01
Ruminococcaceae	*Ruminococcaceae_UCG-005*	-0.45	0.46	5.85E-01	-0.56	0.48	4.76E-01	-1.09	0.60	1.92E-01	-0.35	0.46	7.35E-01
Christensenellaceae	*Christensenellaceae_R-7_group*	-0.39	0.43	6.12E-01	-0.59	0.44	4.00E-01	-1.42	0.56	4.12E-02	-0.37	0.43	6.74E-01
Ruminococcaceae	Unspecified	-0.26	0.29	6.17E-01	-0.14	0.30	8.22E-01	-0.89	0.38	6.43E-02	-0.21	0.30	7.52E-01
Ruminococcaceae	*Ruminococcaceae_NK4A214_group*	-0.40	0.45	6.20E-01	-0.37	0.46	6.57E-01	-1.40	0.58	5.62E-02	-0.39	0.45	6.76E-01
Ruminococcaceae	*Subdoligranulum*	0.31	0.35	6.23E-01	0.31	0.37	6.32E-01	0.64	0.46	3.60E-01	0.32	0.35	6.57E-01
Coriobacteriales_Incertae_Sedis	Unspecified	-0.68	0.77	6.26E-01	-1.02	0.80	4.23E-01	0.00	0.11	9.98E-01	-0.70	0.07	1.84E-21
Lachnospiraceae	*Hungatella*	0.63	0.73	6.27E-01	0.72	0.75	5.76E-01	-0.23	0.95	9.19E-01	0.90	0.75	4.78E-01

Ruminococcaceae	*Faecalibacterium*	-0.25	0.29	6.36E-01	-0.31	0.30	5.34E-01	-0.32	0.38	6.22E-01	-0.30	0.29	5.73E-01
Lactobacillaceae	*Lactobacillus*	-0.40	0.48	6.58E-01	-0.59	0.50	4.63E-01	-0.10	0.63	9.54E-01	-0.19	0.49	8.84E-01
Lachnospiraceae	*Anaerostipes*	0.21	0.26	6.58E-01	0.26	0.27	5.64E-01	0.26	0.34	6.73E-01	0.20	0.26	7.17E-01
Streptococcaceae	*Streptococcus*	-0.26	0.31	6.68E-01	-0.54	0.32	2.45E-01	0.47	0.41	4.69E-01	-0.27	0.31	6.76E-01
Ruminococcaceae	*Ruminiclostridium_9*	-0.28	0.35	6.79E-01	-0.16	0.37	8.26E-01	-0.57	0.46	4.22E-01	-0.25	0.35	7.52E-01
Lachnospiraceae	Unspecified	0.09	0.12	6.95E-01	0.08	0.12	7.48E-01	0.07	0.16	8.34E-01	0.09	0.12	7.52E-01
Acidaminococcaceae	*Phascolarctobacterium*	-0.30	0.43	7.29E-01	-0.30	0.44	7.16E-01	-0.34	0.56	7.50E-01	-0.30	0.43	7.52E-01
Ruminococcaceae	*Ruminiclostridium_6*	0.36	0.55	7.55E-01	-0.04	0.58	9.70E-01	0.48	0.73	7.31E-01	0.36	0.55	7.68E-01
Desulfovibrionaceae	*Desulfovibrio*	0.49	0.86	7.94E-01	-0.64	0.89	6.98E-01	-0.12	1.13	9.68E-01	0.49	0.86	8.08E-01
Prevotellaceae	*Paraprevotella*	-0.47	0.86	8.08E-01	-0.69	0.89	6.67E-01	-1.00	1.13	6.06E-01	-0.40	0.86	8.55E-01
Enterobacteriaceae	*Klebsiella*	-0.38	0.77	8.18E-01	-0.58	0.79	6.88E-01	-0.49	1.00	8.08E-01	-0.38	0.77	8.42E-01
Actinomycetaceae	*Actinomyces*	-0.31	0.61	8.18E-01	-0.71	0.62	4.81E-01	-0.18	0.79	9.26E-01	-0.32	0.60	8.38E-01
Ruminococcaceae	*UBA1819*	0.18	0.35	8.18E-01	0.26	0.37	7.03E-01	-0.09	0.46	9.38E-01	0.27	0.36	7.45E-01
Lachnospiraceae	*Fusicatenibacter*	0.16	0.34	8.26E-01	0.18	0.35	8.00E-01	0.44	0.45	5.52E-01	0.18	0.34	8.38E-01
Ruminococcaceae	*Flavonifractor*	0.19	0.41	8.28E-01	0.25	0.43	7.66E-01	0.14	0.54	9.15E-01	0.21	0.41	8.38E-01
Lachnospiraceae	*Lachnospiraceae_UCG-010*	0.34	0.80	8.40E-01	0.41	0.83	8.06E-01	0.22	1.05	9.29E-01	0.24	0.81	9.19E-01
Family_XIII	*Family_XIII_AD3011_group*	-0.20	0.49	8.41E-01	-0.20	0.50	8.40E-01	-0.49	0.64	6.69E-01	-0.12	0.49	9.36E-01
Christensenellaceae	*Unspecified*	-0.29	0.77	8.56E-01	-0.33	0.80	8.33E-01	-2.34	0.99	6.46E-02	-0.20	0.77	9.32E-01
Bacteroidaceae	*Bacteroides*	-0.07	0.19	8.60E-01	-0.01	0.19	9.70E-01	-0.06	0.24	9.23E-01	-0.07	0.19	8.84E-01
Lachnospiraceae	*Lachnospiraceae_NK4A136_group*	0.11	0.34	8.81E-01	0.03	0.36	9.70E-01	-0.06	0.45	9.67E-01	-0.05	0.35	9.63E-01
Lachnospiraceae	*Roseburia*	-0.11	0.33	8.82E-01	-0.13	0.35	8.53E-01	0.25	0.44	7.61E-01	-0.11	0.34	9.00E-01
Lachnospiraceae	*Coprococcus_3*	-0.15	0.49	8.87E-01	-0.27	0.50	7.88E-01	-0.63	0.64	5.50E-01	-0.19	0.49	8.84E-01
Lachnospiraceae	*Eisenbergiella*	0.17	0.60	8.97E-01	0.16	0.62	9.00E-01	-0.26	0.77	8.80E-01	0.30	0.60	8.42E-01
Ruminococcaceae	*Ruminococcus_1*	0.12	0.43	9.02E-01	0.14	0.45	8.75E-01	-0.58	0.56	5.33E-01	0.14	0.43	9.04E-01
Lachnospiraceae	*Lachnospiraceae_ND3007_group*	0.14	0.50	9.02E-01	0.17	0.52	8.68E-01	0.26	0.66	8.47E-01	0.13	0.02	1.18E-10
Ruminococcaceae	*Negativibacillus*	0.13	0.49	9.09E-01	0.09	0.51	9.39E-01	-0.50	0.64	6.65E-01	0.13	0.49	9.32E-01
Unspecified	Unspecified Clostridia	-0.13	0.58	9.26E-01	-0.10	0.60	9.40E-01	-2.93	0.75	4.47E-04	-0.05	0.59	9.79E-01
Ruminococcaceae	*Angelakisella*	-0.02	0.11	9.56E-01	0.17	0.12	3.53E-01	-1.74	0.23	1.63E-13	-0.17	0.89	9.59E-01
Ruminococcaceae	*Intestinimonas*	-0.01	0.05	9.59E-01	-0.06	0.05	4.51E-01	-1.57	0.69	7.82E-02	0.02	0.05	8.84E-01
Lachnospiraceae	*GCA-900066575*	0.07	0.60	9.61E-01	0.07	0.62	9.61E-01	-0.95	0.78	4.38E-01	0.07	0.60	9.71E-01
Eggerthellaceae	*Eggerthella*	0.07	0.64	9.69E-01	0.14	0.66	9.20E-01	0.14	0.83	9.48E-01	0.02	0.66	9.91E-01
Akkermansiaceae	*Akkermansia*	-0.04	0.36	9.71E-01	-0.05	0.37	9.61E-01	-0.78	0.47	2.46E-01	0.02	0.36	9.84E-01

Lachnospiraceae	*Marvinbryantia*	0.00	0.04	9.78E-01	-0.24	0.04	1.90E-08	0.12	0.05	4.05E-02	0.00	0.04	9.79E-01
Prevotellaceae	*Prevotella_9*	-0.05	0.59	9.78E-01	-0.78	0.61	4.23E-01	0.04	0.77	9.84E-01	-0.05	0.59	9.79E-01
Prevotellaceae	*Prevotella_7*	0.07	1.07	9.84E-01	-0.64	0.05	9.72E-39	-1.42	1.39	5.39E-01	0.01	0.04	9.10E-01
Unspecified	Unspecified Firmitures	0.00	0.07	9.84E-01	-0.27	0.07	2.75E-04	-1.85	0.14	8.16E-38	0.01	0.07	9.59E-01
Ruminococcaceae	*Oscillibacter*	0.01	0.38	9.88E-01	0.10	0.39	8.95E-01	-0.14	0.50	9.08E-01	-0.03	0.38	9.79E-01
Eggerthellaceae	*Adlercreutzia*	0.00	0.09	9.90E-01	-0.03	0.09	8.66E-01	-0.94	0.15	1.07E-09	0.01	0.09	9.62E-01
Peptostreptococcaceae	*Intestinibacter*	NA*	NA	NA	NA	NA	NA	NA	NA	NA	-0.19	0.53	8.87E-01

[a] 10-year exposure window adjusted for sex, race, age, Parkinson's disease status, pesticides co-exposure, and sequencing platform.

[b] 6-to-10 years exposure window, aadjusted for sex, race, age, Parkinson's disease status, pesticides co-exposure, and sequencing platform.

[c] 0-to-5 years exposure window, aadjusted for sex, race, age, Parkinson's disease status, pesticides co-exposure, and sequencing platform.

[d] 10-year exposure window, aadjusted for sex, race, age, pesticides co-exposure, and sequencing platform.

[*] This taxon has NA value due to small cell count therefore excluded from the analysis.

Abbreviations: Log2FC: Log2 Fold Change; SE: Standard Error: Adj: Adjusted.

Table S5. Differential taxa abundance associated with organophosphorus pesticides - Main model and sensitivity analyses - predicted Metacyc pathways (N=190).

Pathway	Main Model[a] (10 year exposure window)			Sensitivity Analyses 1[b] (5 year exposure window - 5 year lag)			Sensitivity Analyses 2[c] (5 year exposure window)			Sensitivity Analyses 3[d] (Excluded PD variable from main model)		
	Log2 FC	SE	Adj P	Log2 FC	SE	Adj P	Log2 FC	SE	Adj P	Log2 FC	SE	Adj P
pyrimidine deoxyribonucleotides de novo biosynthesis IV	0.40	0.12	1.63E-02	0.40	0.13	2.02E-02	0.52	0.16	1.68E-02	0.37	0.13	3.36E-02
methanogenesis from acetate	0.40	0.12	9.86E-03	0.40	0.12	1.26E-02	0.53	0.15	7.68E-03	0.39	0.12	1.10E-02
pyrimidine deoxyribonucleotides biosynthesis from CTP	0.37	0.11	1.51E-02	0.37	0.12	2.27E-02	0.49	0.15	1.34E-02	0.35	0.12	2.92E-02
teichoic acid (poly-glycerol) biosynthesis	0.37	0.09	2.16E-03	0.39	0.10	1.56E-03	0.41	0.12	1.25E-02	0.37	0.09	1.95E-03
dTDP-N-acetylthomosamine biosynthesis	0.36	0.12	3.30E-02	0.36	0.12	3.52E-02	0.49	0.16	1.85E-02	0.36	0.12	2.95E-02
fucose degradation	0.33	0.09	3.40E-03	0.37	0.09	8.50E-04	0.40	0.11	8.47E-03	0.33	0.09	2.86E-03
L-valine degradation I	0.26	0.06	1.05E-04	-0.04	0.05	7.48E-01	0.24	0.07	1.13E-02	0.26	0.06	1.13E-04
superpathway of N-acetylglucosamine, N-acetylmannosamine and N-acetylneuraminate degradation	0.26	0.06	1.15E-03	0.26	0.07	1.79E-03	0.47	0.08	4.47E-07	0.26	0.06	1.36E-03
superpathway of L-alanine biosynthesis	0.24	0.08	3.53E-02	0.23	0.08	4.91E-02	0.44	0.10	4.20E-04	0.24	0.08	3.27E-02
sucrose degradation III (sucrose invertase)	0.21	0.06	1.84E-02	0.23	0.07	6.33E-03	0.36	0.08	3.45E-04	0.20	0.06	2.30E-02
acetylene degradation	0.20	0.06	2.01E-02	0.23	0.06	5.11E-03	0.27	0.08	1.46E-02	0.20	0.06	1.92E-02
peptidoglycan maturation (meso-diaminopimelate containing)	0.20	0.07	3.05E-02	0.23	0.07	1.13E-02	0.29	0.08	7.68E-03	0.20	0.07	2.69E-02
superpathway of pyrimidine deoxyribonucleosides degradation	0.19	0.05	8.22E-03	0.21	0.06	2.55E-03	0.28	0.07	1.64E-03	0.19	0.05	8.81E-03
superpathway of purine deoxyribonucleosides degradation	0.19	0.06	1.91E-02	0.21	0.06	7.05E-03	0.31	0.07	9.65E-04	0.18	0.06	2.01E-02
purine ribonucleosides degradation	0.17	0.06	4.51E-02	0.19	0.06	2.16E-02	0.34	0.08	3.14E-04	0.17	0.06	4.86E-02
L-lysine biosynthesis I	0.14	0.04	1.45E-02	0.17	0.04	3.56E-03	0.20	0.06	6.50E-03	0.14	0.04	1.52E-02
galactose degradation I (Leloir pathway)	0.13	0.04	3.33E-02	0.16	0.05	8.59E-03	0.20	0.06	9.36E-03	0.13	0.05	4.26E-02
pyruvate fermentation to isobutanol (engineered)	0.11	0.04	3.30E-02	0.14	0.04	3.87E-03	0.14	0.05	3.53E-02	0.11	0.04	3.25E-02

L-lysine biosynthesis VI	0.10	0.03	4.72E-02	0.12	0.03	8.82E-03	0.13	0.04	3.56E-02	0.09	0.03	5.81E-02
Calvin-Benson-Bassham cycle	0.09	0.03	4.89E-02	0.10	0.03	1.82E-02	0.13	0.04	2.00E-02	0.09	0.03	5.49E-02
superpathway of thiamin diphosphate biosynthesis II	-0.23	0.08	3.39E-02	-0.26	0.08	1.88E-02	-0.33	0.10	1.46E-02	-0.23	0.08	3.25E-02
chitin derivatives degradation	-0.34	0.04	4.19E-14	-0.39	0.04	5.02E-19	-0.25	0.06	1.59E-03	-0.29	0.04	2.66E-10
creatinine degradation II	-0.36	0.05	1.23E-12	-0.48	0.05	3.16E-21	-0.87	0.07	1.09E-36	-0.34	0.05	1.29E-11
superpathway of histidine, purine, and pyrimidine biosynthesis	-0.37	0.11	1.22E-02	-0.47	0.11	8.89E-04	-0.35	0.15	1.03E-01	-0.37	0.11	1.34E-02
superpathway of pyridoxal 5'-phosphate biosynthesis and salvage	-0.39	0.13	3.38E-02	-0.38	0.14	5.53E-02	-0.49	0.17	4.30E-02	-0.38	0.13	4.36E-02
pyridoxal 5'-phosphate biosynthesis I	-0.41	0.14	3.96E-02	-0.38	0.15	6.70E-02	-0.51	0.19	5.18E-02	-0.39	0.14	5.04E-02
isoprene biosynthesis II (engineered)	-0.45	0.02	2.08E-146	-0.60	0.02	1.33E-247	-0.52	0.02	5.73E-101	-0.11	0.54	9.39E-01
4-aminobutanoate degradation V	-0.51	0.14	5.03E-03	-0.60	0.15	8.81E-04	-0.36	0.19	2.10E-01	-0.52	0.14	3.94E-03
ubiquinol-7 biosynthesis (prokaryotic)	-0.71	0.25	4.83E-02	-0.72	0.26	5.09E-02	-0.77	0.33	1.09E-01	-0.72	0.25	4.13E-02
ubiquinol-9 biosynthesis (prokaryotic)	-0.71	0.25	4.83E-02	-0.72	0.26	5.09E-02	-0.77	0.33	1.09E-01	-0.72	0.25	4.13E-02
ubiquinol-10 biosynthesis (prokaryotic)	-0.71	0.25	4.83E-02	-0.72	0.26	5.09E-02	-0.77	0.33	1.09E-01	-0.72	0.25	4.13E-02
ubiquinol-8 biosynthesis (prokaryotic)	-0.71	0.25	4.83E-02	-0.72	0.26	5.09E-02	-0.77	0.33	1.09E-01	-0.72	0.25	4.13E-02
superpathway of ubiquinol-8 biosynthesis (prokaryotic)	-0.72	0.25	4.50E-02	-0.73	0.26	4.68E-02	-0.77	0.33	1.07E-01	-0.73	0.25	3.84E-02
formaldehyde assimilation I (serine pathway)	-0.94	0.04	1.61E-132	-0.95	0.04	1.25E-124	-1.52	0.06	2.61E-120	-0.94	0.04	6.71E-133
preQ0 biosynthesis	-0.13	0.05	5.11E-02	-0.13	0.05	7.47E-02	-0.19	0.06	2.74E-02	-0.14	0.05	4.24E-02
pentose phosphate pathway (non-oxidative branch)	0.10	0.04	6.03E-02	0.12	0.04	1.40E-02	0.13	0.05	4.71E-02	0.10	0.04	6.47E-02
superpathway of β-D-glucuronide and D-glucuronate degradation	0.20	0.07	6.12E-02	0.21	0.07	4.04E-02	0.36	0.09	2.28E-03	0.18	0.07	8.55E-02
phosphopantothenate biosynthesis I	-0.10	0.04	6.93E-02	-0.07	0.04	2.95E-01	-0.15	0.05	2.72E-02	-0.10	0.04	5.89E-02
glycogen degradation I (bacterial)	0.11	0.04	6.95E-02	0.15	0.04	6.28E-03	0.16	0.05	2.72E-02	0.10	0.04	8.32E-02

superpathway of heme biosynthesis from glycine	-0.85	0.32	7.39E-02	-1.00	0.33	2.69E-02	-0.62	0.42	3.78E-01	-0.84	0.32	6.71E-02
reductive acetyl coenzyme A pathway	-0.62	0.24	7.74E-02	-0.63	0.25	7.28E-02	-0.22	0.31	7.46E-01	-0.62	0.24	7.10E-02
superpathway of glyoxylate bypass and TCA	-0.95	0.36	7.94E-02	-1.09	0.38	3.71E-02	-0.27	0.48	8.11E-01	-1.02	0.36	4.84E-02
superpathway of glycolysis, pyruvate dehydrogenase, TCA, and glyoxylate bypass	-0.91	0.36	8.60E-02	-1.07	0.37	3.73E-02	-0.28	0.47	7.95E-01	-0.98	0.36	5.49E-02
N10-formyl-tetrahydrofolate biosynthesis	0.07	0.03	8.60E-02	0.09	0.03	2.14E-02	0.11	0.04	3.15E-02	0.07	0.03	1.06E-01
purine nucleobases degradation I (anaerobic)	0.23	0.09	8.80E-02	0.27	0.09	3.73E-02	0.33	0.12	4.28E-02	0.24	0.09	7.46E-02
CMP-3-deoxy-D-manno-octulosonate biosynthesis I	-0.28	0.11	8.81E-02	-0.31	0.11	5.38E-02	-0.46	0.14	1.57E-02	-0.28	0.11	7.63E-02
S-adenosyl-L-methionine cycle I	0.12	0.05	8.91E-02	0.14	0.05	3.71E-02	0.19	0.06	1.79E-02	0.11	0.05	1.06E-01
pyruvate fermentation to butanoate	-0.58	0.23	9.74E-02	-0.66	0.24	4.83E-02	-0.55	0.30	2.44E-01	-0.58	0.23	8.55E-02
superpathway of chorismate metabolism	-0.79	0.32	1.01E-01	-0.92	0.33	4.64E-02	-0.42	0.42	5.93E-01	-0.84	0.32	6.80E-02
ADP-L-glycero-β-D-manno-heptose biosynthesis	-0.35	0.14	1.01E-01	-0.51	0.14	5.29E-03	-0.42	0.18	1.22E-01	-0.35	0.14	8.82E-02
glyoxylate cycle	-0.87	0.35	1.01E-01	-0.96	0.36	6.25E-02	-0.36	0.46	7.13E-01	-0.90	0.35	8.07E-02
superpathay of heme biosynthesis from glutamate	-0.50	0.20	1.02E-01	-0.78	0.21	2.87E-03	-0.14	0.27	8.13E-01	-0.50	0.20	8.81E-02
superpathway of Clostridium acetobutylicum acidogenic fermentation	-0.56	0.23	1.03E-01	-0.65	0.24	5.09E-02	-0.54	0.30	2.50E-01	-0.56	0.23	9.18E-02
glycolysis III (from glucose)	0.08	0.03	1.06E-01	0.10	0.03	2.80E-02	0.10	0.04	8.23E-02	0.08	0.03	1.13E-01
heme biosynthesis I (aerobic)	-0.54	0.22	1.06E-01	-0.86	0.22	2.46E-03	-0.10	0.29	8.87E-01	-0.55	0.22	8.81E-02
L-lysine biosynthesis III	0.08	0.03	1.07E-01	0.10	0.03	3.61E-02	0.10	0.04	8.13E-02	0.08	0.03	1.19E-01
superpathway of heme biosynthesis from uroporphyrinogen-III	-0.51	0.21	1.10E-01	-0.83	0.21	2.26E-03	-0.11	0.28	8.62E-01	-0.52	0.21	8.84E-02
Kdo transfer to lipid IVA III (Chlamydia)	-0.31	0.13	1.12E-01	-0.35	0.13	6.23E-02	-0.46	0.16	4.18E-02	-0.31	0.13	9.33E-02
lipid IVA biosynthesis	-0.29	0.12	1.15E-01	-0.32	0.13	7.25E-02	-0.46	0.16	3.39E-02	-0.30	0.12	9.64E-02
L-glutamate and L-glutamine biosynthesis	0.16	0.07	1.16E-01	0.22	0.07	1.97E-02	0.30	0.09	1.13E-02	0.16	0.07	1.29E-01

TCA cycle IV (2-oxoglutarate decarboxylase)	-0.82	0.35	1.33E-01	-0.98	0.37	6.02E-02	-0.35	0.47	7.34E-01	-0.87	0.36	9.64E-02
NAD salvage pathway I	0.09	0.04	1.33E-01	0.12	0.04	2.02E-02	0.13	0.05	6.54E-02	0.08	0.04	1.72E-01
TCA cycle I (prokaryotic)	-0.19	0.08	1.39E-01	-0.21	0.09	7.73E-02	-0.34	0.11	1.70E-02	-0.19	0.08	1.47E-01
L-isoleucine biosynthesis I (from threonine)	0.08	0.03	1.52E-01	0.09	0.04	5.92E-02	0.10	0.04	1.51E-01	0.07	0.03	1.61E-01
L-valine biosynthesis	0.08	0.03	1.52E-01	0.09	0.04	5.92E-02	0.10	0.04	1.51E-01	0.07	0.03	1.61E-01
polyisoprenoid biosynthesis (E. coli)	-0.17	0.07	1.54E-01	-0.19	0.08	9.18E-02	-0.30	0.10	2.13E-02	-0.17	0.07	1.40E-01
phosphatidylglycerol biosynthesis I (plastidic)	0.09	0.04	1.55E-01	0.10	0.04	7.92E-02	0.16	0.05	2.94E-02	0.09	0.04	1.56E-01
phosphatidylglycerol biosynthesis II (non-plastidic)	0.09	0.04	1.55E-01	0.10	0.04	7.92E-02	0.16	0.05	2.94E-02	0.09	0.04	1.56E-01
adenine and adenosine salvage III	0.09	0.04	1.57E-01	0.10	0.04	9.36E-02	0.12	0.05	1.50E-01	0.09	0.04	1.71E-01
chorismate biosynthesis I	0.08	0.03	1.57E-01	0.09	0.04	6.62E-02	0.12	0.05	6.76E-02	0.08	0.04	1.71E-01
superpathway of thiamin diphosphate biosynthesis I	-0.15	0.07	1.59E-01	-0.16	0.07	1.30E-01	-0.26	0.09	3.69E-02	-0.16	0.07	1.39E-01
glycogen biosynthesis I (from ADP-D-Glucose)	0.11	0.05	1.63E-01	0.14	0.05	4.79E-02	0.20	0.06	2.58E-02	0.10	0.05	1.97E-01
superpathway of aromatic amino acid biosynthesis	0.08	0.03	1.64E-01	0.09	0.04	7.13E-02	0.12	0.05	7.09E-02	0.07	0.04	1.82E-01
incomplete reductive TCA cycle	-0.18	0.08	1.65E-01	-0.17	0.09	1.80E-01	-0.35	0.11	1.27E-02	-0.18	0.08	1.56E-01
pyruvate fermentation to acetate and lactate II	0.08	0.04	1.65E-01	0.10	0.04	6.39E-02	0.11	0.05	1.46E-01	0.08	0.04	1.82E-01
superpathway of adenosine nucleotides de novo biosynthesis I	0.07	0.03	1.66E-01	0.07	0.03	1.36E-01	0.11	0.04	5.90E-02	0.07	0.03	1.82E-01
5-aminoimidazole ribonucleotide biosynthesis II	0.07	0.03	1.70E-01	0.08	0.03	6.42E-02	0.07	0.04	2.97E-01	0.07	0.03	1.82E-01
superpathway of 5-aminoimidazole ribonucleotide biosynthesis	0.07	0.03	1.70E-01	0.08	0.03	6.42E-02	0.07	0.04	2.97E-01	0.07	0.03	1.82E-01
superpathway of phospholipid biosynthesis I (bacteria)	0.08	0.04	1.70E-01	0.10	0.04	9.08E-02	0.14	0.05	3.38E-02	0.08	0.04	1.85E-01
L-isoleucine biosynthesis II	0.07	0.03	1.74E-01	0.09	0.04	7.13E-02	0.09	0.05	1.65E-01	0.07	0.03	1.93E-01
pantothenate and coenzyme A biosynthesis I	-0.07	0.03	1.75E-01	-0.04	0.03	4.49E-01	-0.09	0.04	1.30E-01	-0.07	0.03	1.57E-01

superpathway of 2,3-butanediol biosynthesis	0.47	0.22	1.75E-01	0.25	0.23	5.25E-01	0.84	0.28	3.06E-02	0.35	0.22	3.75E-01
superpathway of L-serine and glycine biosynthesis I	0.08	0.04	1.79E-01	0.11	0.04	5.08E-02	0.12	0.05	1.11E-01	0.08	0.04	2.04E-01
superpathway of adenosine nucleotides de novo biosynthesis II	0.07	0.03	1.81E-01	0.07	0.03	1.86E-01	0.11	0.04	6.92E-02	0.07	0.03	1.97E-01
urea cycle	-0.35	0.16	1.84E-01	-0.48	0.17	4.13E-02	-0.36	0.21	2.88E-01	-0.33	0.16	2.05E-01
UDP-N-acetylmuramoyl-pentapeptide biosynthesis I (meso-diaminopimelate containing)	0.07	0.03	1.84E-01	0.08	0.03	9.20E-02	0.09	0.04	1.62E-01	0.07	0.03	2.08E-01
L-isoleucine biosynthesis III	0.07	0.04	1.84E-01	0.09	0.04	7.29E-02	0.09	0.05	2.12E-01	0.07	0.04	2.06E-01
peptidoglycan biosynthesis I (meso-diaminopimelate containing)	0.07	0.03	1.85E-01	0.08	0.03	9.15E-02	0.09	0.04	1.58E-01	0.07	0.03	2.09E-01
superpathway of L-isoleucine biosynthesis I	0.06	0.03	1.88E-01	0.08	0.03	7.26E-02	0.07	0.04	2.88E-01	0.06	0.03	2.24E-01
adenosine deoxyribonucleotides de novo biosynthesis II	0.09	0.04	1.88E-01	0.07	0.05	3.40E-01	0.16	0.06	4.55E-02	0.09	0.04	1.93E-01
guanosine deoxyribonucleotides de novo biosynthesis II	0.09	0.04	1.88E-01	0.07	0.05	3.40E-01	0.16	0.06	4.55E-02	0.09	0.04	1.93E-01
pyruvate fermentation to propanoate I	-0.22	0.11	1.95E-01	-0.19	0.11	2.57E-01	-0.41	0.14	2.85E-02	-0.22	0.11	1.86E-01
UDP-N-acetylmuramoyl-pentapeptide biosynthesis II (lysine-containing)	0.07	0.03	1.99E-01	0.08	0.04	9.96E-02	0.09	0.04	1.62E-01	0.07	0.03	2.26E-01
sulfate reduction I (assimilatory)	-0.22	0.11	1.99E-01	-0.30	0.11	6.12E-02	-0.32	0.14	1.41E-01	-0.22	0.11	2.07E-01
UMP biosynthesis	0.07	0.03	1.99E-01	0.09	0.03	5.28E-02	0.09	0.04	1.56E-01	0.07	0.03	2.21E-01
pyrimidine deoxyribonucleotide phosphorylation	-0.16	0.08	1.99E-01	-0.21	0.08	5.50E-02	-0.29	0.10	3.13E-02	-0.15	0.08	2.03E-01
superpathway of branched amino acid biosynthesis	0.07	0.03	2.01E-01	0.09	0.04	8.65E-02	0.08	0.05	2.68E-01	0.07	0.03	2.23E-01
glutaryl-CoA degradation	-0.49	0.24	2.01E-01	-0.58	0.25	9.96E-02	-0.42	0.31	4.25E-01	-0.48	0.24	2.07E-01
starch degradation V	0.09	0.04	2.01E-01	0.12	0.05	6.02E-02	0.12	0.06	1.64E-01	0.08	0.05	2.54E-01
guanosine ribonucleotides de novo biosynthesis	0.06	0.03	2.01E-01	0.08	0.03	7.34E-02	0.08	0.04	1.81E-01	0.06	0.03	2.24E-01
tRNA charging	0.07	0.03	2.01E-01	0.08	0.03	9.23E-02	0.08	0.04	1.97E-01	0.06	0.03	2.25E-01
peptidoglycan biosynthesis III (mycobacteria)	0.07	0.03	2.01E-01	0.08	0.03	9.96E-02	0.09	0.04	1.60E-01	0.06	0.03	2.28E-01

anhydromuropeptides recycling	-0.24	0.12	2.09E-01	-0.32	0.12	5.92E-02	-0.25	0.15	3.14E-01	-0.23	0.12	2.21E-01
thiamin salvage II	0.07	0.04	2.09E-01	0.09	0.04	9.09E-02	0.08	0.05	2.83E-01	0.08	0.04	2.05E-01
TCA cycle V (2-oxoglutarate:ferredoxin oxidoreductase)	-0.16	0.08	2.09E-01	-0.16	0.08	2.00E-01	-0.33	0.10	1.84E-02	-0.16	0.08	2.09E-01
TCA cycle VI (obligate autotrophs)	-0.21	0.10	2.10E-01	-0.27	0.11	8.69E-02	-0.38	0.14	4.71E-02	-0.21	0.11	2.23E-01
chorismate biosynthesis from 3-dehydroquinate	0.07	0.03	2.10E-01	0.08	0.03	9.23E-02	0.11	0.04	7.66E-02	0.06	0.03	2.42E-01
arginine, ornithine and proline interconversion	-0.28	0.14	2.11E-01	-0.31	0.15	1.46E-01	-0.30	0.19	3.26E-01	-0.27	0.15	2.87E-01
sucrose degradation IV (sucrose phosphorylase)	0.18	0.09	2.11E-01	0.22	0.10	9.96E-02	0.48	0.12	1.02E-03	0.18	0.09	2.17E-01
superpathway of menaquinol-9 biosynthesis	-0.48	0.24	2.11E-01	-0.60	0.25	9.49E-02	-0.33	0.32	5.87E-01	-0.50	0.24	1.93E-01
superpathway of menaquinol-6 biosynthesis I	-0.48	0.24	2.11E-01	-0.60	0.25	9.49E-02	-0.33	0.32	5.87E-01	-0.50	0.24	1.93E-01
superpathway of menaquinol-10 biosynthesis	-0.48	0.24	2.11E-01	-0.60	0.25	9.49E-02	-0.33	0.32	5.87E-01	-0.50	0.24	1.93E-01
superpathway of sulfate assimilation and cysteine biosynthesis	-0.18	0.09	2.11E-01	-0.24	0.09	7.58E-02	-0.27	0.12	1.23E-01	-0.18	0.09	2.24E-01
fatty acid β-oxidation I	-0.52	0.26	2.13E-01	-0.60	0.27	1.35E-01	-0.20	0.35	8.05E-01	-0.57	0.27	1.69E-01
superpathway of pyrimidine nucleobases salvage	0.06	0.03	2.19E-01	0.08	0.03	1.20E-01	0.08	0.04	2.06E-01	0.06	0.03	2.47E-01
superpathway of hexuronide and hexuronate degradation	0.14	0.07	2.20E-01	0.16	0.07	1.13E-01	0.22	0.09	8.49E-02	0.12	0.07	2.97E-01
CDP-diacylglycerol biosynthesis I	0.07	0.04	2.24E-01	0.08	0.04	1.32E-01	0.13	0.05	4.71E-02	0.07	0.04	2.45E-01
CDP-diacylglycerol biosynthesis II	0.07	0.04	2.24E-01	0.08	0.04	1.32E-01	0.13	0.05	4.71E-02	0.07	0.04	2.45E-01
superpathway of fucose and rhamnose degradation	0.14	0.07	2.25E-01	0.20	0.08	5.69E-02	0.08	0.10	7.15E-01	0.14	0.07	2.27E-01
L-arginine biosynthesis I (via L-ornithine)	0.07	0.04	2.26E-01	0.10	0.04	8.74E-02	0.08	0.05	3.08E-01	0.08	0.04	2.24E-01
enterobactin biosynthesis	-0.69	0.35	2.26E-01	-0.83	0.37	1.20E-01	-0.20	0.47	8.59E-01	-0.74	0.35	1.93E-01
L-arginine biosynthesis IV (archaebacteria)	0.07	0.04	2.26E-01	0.10	0.04	8.69E-02	0.08	0.05	3.13E-01	0.07	0.04	2.24E-01
5-aminoimidazole ribonucleotide biosynthesis I	0.06	0.03	2.28E-01	0.07	0.03	9.18E-02	0.06	0.04	3.67E-01	0.05	0.03	2.54E-01

L-arginine biosynthesis II (acetyl cycle)	0.09	0.04	2.29E-01	0.11	0.05	7.66E-02	0.09	0.06	3.37E-01	0.09	0.04	2.26E-01
L-arginine degradation II (AST pathway)	-0.76	0.40	2.38E-01	-0.89	0.41	1.38E-01	0.11	0.52	9.38E-01	-0.92	0.40	1.27E-01
glycolysis V (Pyrococcus)	1.05	0.55	2.40E-01	1.04	0.57	2.34E-01	0.86	0.72	5.07E-01	0.99	0.55	2.84E-01
pyrimidine deoxyribonucleotides de novo biosynthesis III	-0.12	0.06	2.41E-01	-0.15	0.06	1.07E-01	-0.29	0.08	4.10E-03	-0.12	0.06	2.35E-01
pyrimidine deoxyribonucleotides de novo biosynthesis I	-0.13	0.07	2.41E-01	-0.19	0.07	6.30E-02	-0.23	0.09	6.92E-02	-0.13	0.07	2.50E-01
GDP-D-glycero-α-D-manno-heptose biosynthesis	0.22	0.12	2.44E-01	0.19	0.12	3.29E-01	0.34	0.15	1.37E-01	0.23	0.12	2.43E-01
superpathway of L-threonine biosynthesis	0.06	0.03	2.44E-01	0.07	0.03	9.96E-02	0.05	0.04	4.03E-01	0.05	0.03	3.06E-01
superpathway of guanosine nucleotides de novo biosynthesis I	-0.13	0.07	2.53E-01	-0.17	0.07	9.96E-02	-0.24	0.09	5.97E-02	-0.13	0.07	2.50E-01
superpathway of (Kdo)2-lipid A biosynthesis	-0.63	0.34	2.55E-01	-0.96	0.35	5.09E-02	-0.46	0.44	5.80E-01	-0.62	0.34	2.67E-01
isopropanol biosynthesis	-0.70	0.37	2.55E-01	-0.89	0.39	1.07E-01	-0.60	0.49	4.88E-01	-0.71	0.37	2.41E-01
queuosine biosynthesis	-0.10	0.05	2.55E-01	-0.10	0.06	2.75E-01	-0.10	0.07	4.03E-01	-0.11	0.05	2.27E-01
superpathway of demethylmenaquinol-6 biosynthesis I	-0.46	0.25	2.60E-01	-0.58	0.26	1.24E-01	-0.26	0.33	7.17E-01	-0.49	0.25	2.24E-01
superpathway of demethylmenaquinol-9 biosynthesis	-0.46	0.25	2.60E-01	-0.58	0.26	1.24E-01	-0.26	0.33	7.17E-01	-0.49	0.25	2.24E-01
superpathway of L-methionine biosynthesis (by sulfhydrylation)	-0.31	0.17	2.66E-01	-0.46	0.17	6.57E-02	-0.55	0.22	8.42E-02	-0.30	0.17	3.04E-01
superpathway of pyrimidine ribonucleotides de novo biosynthesis	-0.11	0.06	2.66E-01	-0.14	0.06	1.38E-01	-0.22	0.08	4.37E-02	-0.11	0.06	2.60E-01
tRNA processing	-0.33	0.19	2.83E-01	-0.53	0.19	4.83E-02	-0.12	0.24	8.30E-01	-0.34	0.19	2.72E-01
L-arginine biosynthesis III (via N-acetyl-L-citrulline)	-0.20	0.11	2.90E-01	-0.16	0.11	3.72E-01	-0.34	0.14	1.09E-01	-0.20	0.11	2.80E-01
superpathway of L-lysine, L-threonine and L-methionine biosynthesis I	0.11	0.06	2.95E-01	0.06	0.07	6.01E-01	0.23	0.08	4.70E-02	0.11	0.06	2.96E-01
biotin biosynthesis II	-0.44	0.25	2.95E-01	-0.54	0.26	1.58E-01	-0.63	0.33	2.12E-01	-0.41	0.25	3.38E-01
superpathway of menaquinol-8 biosynthesis II	-0.39	0.22	2.95E-01	-0.40	0.23	2.59E-01	-0.90	0.29	1.90E-02	-0.38	0.22	3.13E-01
superpathway of menaquinol-8 biosynthesis I	-0.33	0.19	2.98E-01	-0.50	0.19	6.87E-02	-0.67	0.25	5.32E-02	-0.33	0.19	3.15E-01

methylphosphonate degradation I	-0.35	0.20	2.98E-01	-0.38	0.20	2.26E-01	-0.39	0.26	3.58E-01	-0.35	0.20	2.99E-01
superpathway of geranylgeranyl diphosphate biosynthesis II (via MEP)	0.06	0.04	2.98E-01	0.09	0.04	1.03E-01	0.06	0.05	4.37E-01	0.06	0.04	3.55E-01
superpathway of purine nucleotides de novo biosynthesis I	-0.10	0.05	2.98E-01	-0.12	0.06	1.46E-01	-0.20	0.07	4.70E-02	-0.10	0.05	2.97E-01
adenosine ribonucleotides de novo biosynthesis	0.06	0.03	2.98E-01	0.07	0.03	1.41E-01	0.08	0.04	2.29E-01	0.05	0.03	3.32E-01
colanic acid building blocks biosynthesis	-0.12	0.07	2.99E-01	-0.12	0.07	2.82E-01	-0.29	0.09	1.22E-02	-0.12	0.07	2.96E-01
superpathway of guanosine nucleotides de novo biosynthesis II	-0.12	0.07	3.01E-01	-0.15	0.07	1.27E-01	-0.22	0.09	6.97E-02	-0.12	0.07	3.05E-01
L-isoleucine biosynthesis IV	0.06	0.04	3.04E-01	0.09	0.04	1.26E-01	0.08	0.05	3.50E-01	0.06	0.04	3.30E-01
superpathway of menaquinol-7 biosynthesis	-0.32	0.19	3.10E-01	-0.50	0.19	7.27E-02	-0.66	0.25	5.90E-02	-0.32	0.19	3.30E-01
superpathway of L-methionine biosynthesis (transsulfuration)	0.11	0.06	3.11E-01	0.10	0.06	3.16E-01	0.19	0.08	1.16E-01	0.11	0.06	3.10E-01
superpathway of GDP-mannose-derived O-antigen building blocks biosynthesis	-0.15	0.09	3.13E-01	-0.14	0.09	3.33E-01	-0.35	0.11	1.65E-02	-0.15	0.09	2.94E-01
superpathway of menaquinol-11 biosynthesis	-0.33	0.19	3.13E-01	-0.51	0.20	7.13E-02	-0.65	0.25	6.76E-02	-0.33	0.20	3.30E-01
superpathway of menaquinol-12 biosynthesis	-0.33	0.19	3.13E-01	-0.51	0.20	7.13E-02	-0.65	0.25	6.76E-02	-0.33	0.20	3.30E-01
superpathway of menaquinol-13 biosynthesis	-0.33	0.19	3.13E-01	-0.51	0.20	7.13E-02	-0.65	0.25	6.76E-02	-0.33	0.20	3.30E-01
polymyxin resistance	-0.63	0.38	3.18E-01	-0.74	0.39	2.10E-01	0.05	0.49	9.67E-01	-0.76	0.38	2.10E-01
fatty acid elongation -- saturated	-0.17	0.10	3.21E-01	-0.21	0.10	1.92E-01	-0.35	0.13	5.73E-02	-0.18	0.10	3.06E-01
peptidoglycan biosynthesis II (staphylococci)	0.64	0.38	3.21E-01	0.47	0.39	4.69E-01	0.66	0.50	4.34E-01	0.60	0.38	3.77E-01
superpathway of pyrimidine ribonucleosides salvage	-0.12	0.07	3.21E-01	-0.15	0.07	1.51E-01	-0.24	0.09	5.80E-02	-0.12	0.07	3.19E-01
fatty acid salvage	-1.23	0.75	3.33E-01	-1.33	0.78	2.73E-01	-0.66	0.99	7.63E-01	-1.23	0.75	3.45E-01
catechol degradation to β-ketoadipate	-0.93	0.57	3.44E-01	-1.25	0.59	1.50E-01	-0.61	0.75	7.06E-01	-1.18	0.58	1.95E-01
protocatechuate degradation II (ortho-cleavage pathway)	-0.92	0.57	3.44E-01	-1.35	0.59	1.09E-01	-0.63	0.75	6.86E-01	-1.08	0.57	2.43E-01
inosine-5'-phosphate biosynthesis I	0.05	0.03	3.49E-01	0.06	0.03	1.68E-01	0.05	0.04	4.42E-01	0.04	0.03	4.06E-01

L-histidine biosynthesis	0.06	0.04	3.50E-01	0.08	0.04	1.39E-01	0.08	0.05	3.54E-01	0.06	0.04	3.93E-01
dTDP-L-rhamnose biosynthesis I	0.06	0.04	3.58E-01	0.09	0.04	9.08E-02	0.11	0.05	1.22E-01	0.06	0.04	3.97E-01
aerobic respiration I (cytochrome c)	-0.67	0.43	3.58E-01	-0.79	0.44	2.44E-01	-0.50	0.56	6.65E-01	-0.68	0.43	3.65E-01
superpathway of demethylmenaquinol-8 biosynthesis	-0.32	0.20	3.59E-01	-0.53	0.21	7.28E-02	-0.62	0.26	1.03E-01	-0.32	0.20	3.76E-01
peptidoglycan biosynthesis IV (Enterococcus faecium)	0.14	0.09	3.59E-01	0.14	0.09	3.40E-01	0.33	0.12	4.13E-02	0.14	0.09	3.93E-01
1,4-dihydroxy-6-naphthoate biosynthesis II	-0.37	0.23	3.60E-01	-0.37	0.24	3.29E-01	-0.94	0.30	1.93E-02	-0.36	0.23	3.92E-01
allantoin degradation to glyoxylate III	0.24	0.15	3.61E-01	0.29	0.16	2.17E-01	0.28	0.20	4.03E-01	0.22	0.16	4.35E-01
methylerythritol phosphate pathway I	0.06	0.04	3.70E-01	0.08	0.04	1.35E-01	0.05	0.05	5.47E-01	0.05	0.04	4.34E-01
methylerythritol phosphate pathway II	0.06	0.04	3.70E-01	0.08	0.04	1.35E-01	0.05	0.05	5.47E-01	0.05	0.04	4.34E-01
adenosylcobalamin salvage from cobinamide II	0.08	0.05	3.74E-01	0.11	0.05	1.81E-01	0.13	0.07	2.30E-01	0.07	0.05	4.38E-01
adenosylcobalamin biosynthesis from cobyrinate a,c-diamide I	0.08	0.05	3.76E-01	0.11	0.05	1.81E-01	0.13	0.07	2.35E-01	0.07	0.05	4.40E-01
superpathway of glycol metabolism and degradation	-0.50	0.33	3.76E-01	-0.58	0.34	2.68E-01	-0.27	0.43	7.81E-01	-0.51	0.33	3.82E-01
Bifidobacterium shunt	0.25	0.17	3.76E-01	0.28	0.17	3.07E-01	0.31	0.22	3.94E-01	0.22	0.17	4.85E-01
L-lysine biosynthesis II	0.28	0.18	3.76E-01	0.11	0.19	7.90E-01	0.46	0.24	2.17E-01	0.27	0.18	4.11E-01
catechol degradation III (ortho-cleavage pathway)	-0.89	0.58	3.76E-01	-1.24	0.60	1.62E-01	-0.57	0.76	7.34E-01	-1.18	0.59	2.09E-01
aromatic compounds degradation via β-ketoadipate	-0.89	0.58	3.76E-01	-1.24	0.60	1.62E-01	-0.57	0.76	7.34E-01	-1.18	0.59	2.09E-01
superpathway of purine nucleotides de novo biosynthesis II	-0.08	0.05	3.79E-01	-0.10	0.05	2.10E-01	-0.18	0.07	6.38E-02	-0.08	0.05	3.92E-01
L-ornithine biosynthesis	0.07	0.04	3.82E-01	0.11	0.04	9.96E-02	0.05	0.06	6.84E-01	0.07	0.04	3.93E-01
UDP-2,3-diacetamido-2,3-dideoxy-α-D-mannuronate biosynthesis	-0.34	0.23	3.98E-01	-0.35	0.24	3.54E-01	-0.59	0.30	2.04E-01	-0.34	0.23	4.08E-01
superpathway of polyamine biosynthesis II	-0.17	0.11	3.98E-01	-0.26	0.12	1.30E-01	0.08	0.15	8.31E-01	-0.17	0.11	4.17E-01
1,4-dihydroxy-2-naphthoate biosynthesis I	-0.33	0.22	3.98E-01	-0.60	0.23	6.72E-02	-0.54	0.29	2.45E-01	-0.35	0.23	3.91E-01

adenosylcobalamin salvage from cobinamide I	0.08	0.05	4.00E-01	0.10	0.05	2.10E-01	0.12	0.07	2.68E-01	0.07	0.05	4.74E-01
superpathway of phylloquinol biosynthesis	-0.32	0.22	4.03E-01	-0.59	0.23	7.27E-02	-0.54	0.29	2.33E-01	-0.34	0.23	3.95E-01
superpathway of S-adenosyl-L-methionine biosynthesis	0.11	0.07	4.03E-01	0.09	0.08	4.86E-01	0.23	0.10	1.04E-01	0.11	0.08	4.18E-01
L-methionine biosynthesis I	0.12	0.08	4.05E-01	0.10	0.09	5.10E-01	0.26	0.11	1.04E-01	0.12	0.08	4.26E-01
superpathway of pyrimidine deoxyribonucleotides de novo biosynthesis	-0.10	0.07	4.07E-01	-0.11	0.07	2.95E-01	-0.09	0.09	5.77E-01	-0.09	0.07	4.40E-01
superpathway of pyrimidine deoxyribonucleoside salvage	-0.08	0.06	4.07E-01	-0.13	0.06	1.50E-01	-0.19	0.07	7.09E-02	-0.08	0.06	4.31E-01
superpathway of salicylate degradation	-0.87	0.62	4.11E-01	-1.22	0.64	2.04E-01	-0.55	0.81	7.58E-01	-1.18	0.62	2.41E-01
superpathway of UDP-glucose-derived O-antigen building blocks biosynthesis	-0.23	0.16	4.16E-01	-0.44	0.17	6.42E-02	-0.24	0.21	5.38E-01	-0.26	0.17	3.67E-01
aromatic biogenic amine degradation (bacteria)	0.53	0.38	4.16E-01	0.50	0.39	4.29E-01	-0.89	0.50	2.61E-01	0.47	0.38	5.12E-01
toluene degradation III (aerobic) (via p-cresol)	-0.89	0.64	4.21E-01	-1.22	0.67	2.33E-01	-0.58	0.85	7.58E-01	-1.11	0.65	3.13E-01
reductive TCA cycle I	-0.26	0.19	4.28E-01	-0.43	0.19	1.29E-01	-0.05	0.25	9.40E-01	-0.26	0.19	4.43E-01
L-leucine degradation I	-1.11	0.81	4.32E-01	-1.14	0.84	3.96E-01	-0.85	1.09	7.16E-01	-1.18	0.81	4.17E-01
urate biosynthesis/inosine 5'-phosphate degradation	-0.08	0.06	4.32E-01	-0.10	0.06	2.50E-01	-0.21	0.07	4.04E-02	-0.08	0.06	4.68E-01
chondroitin sulfate degradation I (bacterial)	-0.26	0.19	4.36E-01	-0.20	0.20	5.47E-01	-0.24	0.25	6.35E-01	-0.27	0.19	4.32E-01
L-histidine degradation I	-0.17	0.13	4.39E-01	-0.12	0.13	6.12E-01	-0.37	0.16	1.23E-01	-0.17	0.13	4.73E-01
4-hydroxyphenylacetate degradation	-0.80	0.59	4.43E-01	-1.04	0.62	2.75E-01	-0.34	0.78	8.54E-01	-1.17	0.60	2.24E-01
4-methylcatechol degradation (ortho cleavage)	-0.86	0.64	4.48E-01	-1.19	0.66	2.44E-01	-0.58	0.84	7.53E-01	-1.11	0.64	3.11E-01
biotin biosynthesis I	-0.16	0.12	4.66E-01	-0.16	0.13	4.48E-01	-0.33	0.16	1.64E-01	-0.17	0.12	4.42E-01
superpathway of pyrimidine deoxyribonucleotides de novo biosynthesis (E. coli)	-0.07	0.05	4.66E-01	-0.11	0.05	1.68E-01	-0.16	0.07	9.57E-02	-0.06	0.05	5.02E-01
enterobacterial common antigen biosynthesis	-0.50	0.39	4.67E-01	-0.62	0.40	3.29E-01	0.00	0.51	1.00E+00	-0.55	0.39	4.38E-01
mannan degradation	-0.15	0.11	4.70E-01	-0.11	0.12	5.84E-01	-0.40	0.15	5.11E-02	-0.16	0.11	4.48E-01

superpathway of arginine and polyamine biosynthesis	-0.13	0.10	4.83E-01	-0.19	0.11	2.45E-01	-0.06	0.14	8.55E-01	-0.13	0.10	5.04E-01
flavin biosynthesis I (bacteria and plants)	-0.04	0.03	4.85E-01	-0.02	0.03	6.75E-01	-0.03	0.04	7.63E-01	-0.04	0.03	4.69E-01
heterolactic fermentation	0.19	0.15	4.90E-01	0.22	0.16	4.04E-01	0.31	0.20	3.60E-01	0.17	0.16	5.78E-01
8-amino-7-oxononanoate biosynthesis I	-0.16	0.13	4.92E-01	-0.16	0.14	4.85E-01	-0.36	0.17	1.60E-01	-0.18	0.13	4.70E-01
ketogluconate metabolism	-0.50	0.41	4.94E-01	-0.86	0.42	1.68E-01	-0.11	0.54	9.38E-01	-0.50	0.41	5.12E-01
lactose and galactose degradation I	0.30	0.25	5.00E-01	0.05	0.26	9.31E-01	0.85	0.32	6.09E-02	0.29	0.25	5.43E-01
D-fructuronate degradation	0.07	0.06	5.02E-01	0.12	0.06	1.92E-01	0.04	0.08	8.31E-01	0.07	0.06	5.63E-01
mixed acid fermentation	-0.08	0.06	5.06E-01	-0.07	0.07	5.67E-01	-0.20	0.08	9.67E-02	-0.08	0.07	5.02E-01
adenosylcobalamin biosynthesis I (early cobalt insertion)	-1.13	0.96	5.18E-01	-1.20	0.99	4.58E-01	-1.31	1.26	5.80E-01	-0.55	0.01	#######
norspermidine biosynthesis	-0.78	0.66	5.18E-01	-1.13	0.68	2.84E-01	-0.40	0.86	8.42E-01	-1.20	0.66	2.78E-01
allantoin degradation IV (anaerobic)	0.55	0.46	5.20E-01	0.61	0.48	4.33E-01	0.56	0.61	6.52E-01	0.57	0.47	5.16E-01
palmitate biosynthesis II (bacteria and plants)	-0.17	0.14	5.21E-01	-0.25	0.15	2.80E-01	-0.52	0.19	4.73E-02	-0.17	0.14	5.29E-01
superpathway of polyamine biosynthesis I	-0.14	0.12	5.27E-01	-0.22	0.12	2.50E-01	-0.01	0.16	9.84E-01	-0.14	0.12	5.52E-01
coenzyme A biosynthesis I	0.04	0.03	5.34E-01	0.05	0.04	3.74E-01	0.07	0.04	2.94E-01	0.04	0.03	5.71E-01
O-antigen building blocks biosynthesis (E. coli)	0.06	0.05	5.36E-01	0.06	0.05	4.57E-01	0.08	0.07	5.16E-01	0.06	0.05	5.61E-01
1,4-dihydroxy-6-naphthoate biosynthesis I	-0.36	0.32	5.39E-01	-0.39	0.33	4.69E-01	-0.65	0.42	3.36E-01	-0.35	0.32	5.63E-01
heme biosynthesis II (anaerobic)	-0.20	0.18	5.53E-01	-0.45	0.18	8.89E-02	0.16	0.23	7.56E-01	-0.22	0.18	5.22E-01
adenosine nucleotides degradation IV	-0.49	0.44	5.54E-01	-0.59	0.46	4.27E-01	0.15	0.58	9.16E-01	-0.44	0.44	5.97E-01
guanosine nucleotides degradation III	0.07	0.07	5.55E-01	0.10	0.07	3.91E-01	0.06	0.09	7.58E-01	0.08	0.07	5.29E-01
superpathway of L-aspartate and L-asparagine biosynthesis	0.04	0.04	5.55E-01	0.05	0.04	4.27E-01	0.05	0.05	5.77E-01	0.03	0.04	6.37E-01
myo-inositol degradation I	0.17	0.16	5.55E-01	0.23	0.16	3.74E-01	0.30	0.20	3.58E-01	0.17	0.16	5.63E-01

superpathway of methylglyoxal degradation	-0.41	0.38	5.58E-01	-0.46	0.39	4.81E-01	-0.01	0.50	9.96E-01	-0.46	0.38	5.21E-01
NAD biosynthesis I (from aspartate)	0.04	0.04	5.59E-01	0.09	0.04	1.37E-01	0.04	0.05	6.68E-01	0.04	0.04	6.05E-01
superpathway of ornithine degradation	-0.42	0.39	5.62E-01	-0.55	0.41	3.96E-01	0.06	0.52	9.63E-01	-0.47	0.39	5.29E-01
succinate fermentation to butanoate	-0.14	0.13	5.82E-01	-0.19	0.14	3.92E-01	-0.19	0.18	5.47E-01	-0.14	0.13	5.89E-01
pyrimidine deoxyribonucleosides salvage	0.04	0.04	5.86E-01	0.03	0.04	6.70E-01	0.05	0.06	6.64E-01	0.04	0.04	6.43E-01
adenosine nucleotides degradation II	0.09	0.09	5.93E-01	0.14	0.09	3.40E-01	0.03	0.11	9.05E-01	0.09	0.09	5.67E-01
superpathway of L-arginine, putrescine, and 4-aminobutanoate degradation	-0.40	0.40	5.93E-01	-0.47	0.41	4.99E-01	0.06	0.52	9.65E-01	-0.45	0.40	5.43E-01
superpathway of L-arginine and L-ornithine degradation	-0.40	0.40	5.93E-01	-0.47	0.41	4.99E-01	0.06	0.52	9.65E-01	-0.45	0.40	5.43E-01
superpathway of glycerol degradation to 1,3-propanediol	0.27	0.27	6.03E-01	0.07	0.28	9.26E-01	0.48	0.36	4.30E-01	0.27	0.27	5.90E-01
mycolate biosynthesis	-0.13	0.13	6.18E-01	-0.17	0.13	4.53E-01	-0.36	0.17	1.54E-01	-0.14	0.13	5.69E-01
(5Z)-dodec-5-enoate biosynthesis	-0.13	0.13	6.22E-01	-0.18	0.14	4.32E-01	-0.36	0.17	1.76E-01	-0.14	0.14	5.78E-01
L-1,2-propanediol degradation	0.21	0.22	6.26E-01	0.17	0.23	7.15E-01	-0.09	0.29	9.02E-01	0.21	0.22	6.17E-01
oleate biosynthesis IV (anaerobic)	-0.12	0.13	6.30E-01	-0.16	0.14	4.58E-01	-0.36	0.17	1.60E-01	-0.14	0.13	5.78E-01
L-methionine biosynthesis III	0.08	0.09	6.48E-01	0.05	0.09	8.00E-01	0.20	0.12	2.74E-01	0.08	0.09	6.51E-01
stearate biosynthesis II (bacteria and plants)	-0.12	0.14	6.48E-01	-0.16	0.14	4.92E-01	-0.38	0.18	1.62E-01	-0.14	0.14	5.88E-01
superpathway of fatty acid biosynthesis initiation (E. coli)	-0.12	0.14	6.48E-01	-0.17	0.14	4.57E-01	-0.36	0.18	1.73E-01	-0.14	0.14	5.92E-01
gluconeogenesis I	-0.03	0.04	6.48E-01	-0.02	0.04	8.32E-01	-0.10	0.05	1.68E-01	-0.03	0.04	6.26E-01
6-hydroxymethyl-dihydropterin diphosphate biosynthesis III (Chlamydia)	-0.04	0.04	6.48E-01	-0.03	0.05	6.94E-01	-0.01	0.06	9.20E-01	-0.04	0.04	6.01E-01
purine nucleotides degradation II (aerobic)	0.06	0.06	6.52E-01	0.09	0.07	3.95E-01	0.02	0.08	9.37E-01	0.06	0.06	6.08E-01
factor 420 biosynthesis	-0.54	0.61	6.54E-01	-0.58	0.63	6.05E-01	-1.67	0.79	1.62E-01	-0.36	0.61	8.09E-01
TCA cycle VII (acetate-producers)	-0.16	0.18	6.54E-01	-0.30	0.19	3.05E-01	0.08	0.24	8.82E-01	-0.17	0.18	6.19E-01

palmitoleate biosynthesis I (from (5Z)-dodec-5-enoate)	-0.12	0.14	6.55E-01	-0.17	0.14	4.83E-01	-0.37	0.18	1.64E-01	-0.14	0.14	5.96E-01
L-glutamate degradation VIII (to propanoate)	-0.95	1.08	6.58E-01	-0.54	0.04	3.45E-44	-0.43	0.04	2.39E-20	-0.68	0.04	1.31E-78
7-(3-amino-3-carboxypropyl)-wyosine biosynthesis	-0.54	0.62	6.60E-01	-0.57	0.64	6.16E-01	-1.70	0.80	1.59E-01	-0.37	0.62	8.08E-01
glucose and glucose-1-phosphate degradation	-0.21	0.24	6.62E-01	-0.47	0.25	2.17E-01	-0.19	0.32	7.96E-01	-0.30	0.25	5.22E-01
methanogenesis from H2 and CO2	-0.53	0.62	6.65E-01	-0.57	0.64	6.20E-01	-1.67	0.81	1.68E-01	-0.36	0.63	8.13E-01
archaetidylserine and archaetidylethanolamine biosynthesis	-0.52	0.61	6.69E-01	-0.56	0.63	6.25E-01	-1.68	0.79	1.61E-01	-0.36	0.62	8.13E-01
chorismate biosynthesis II (archaea)	-0.36	0.42	6.69E-01	-0.41	0.44	5.91E-01	-0.56	0.55	5.87E-01	-0.34	0.42	6.96E-01
archaetidylinositol biosynthesis	-0.51	0.61	6.73E-01	-0.55	0.63	6.31E-01	-1.67	0.79	1.64E-01	-0.36	0.62	8.13E-01
TCA cycle VIII (helicobacter)	-0.15	0.18	6.78E-01	-0.15	0.19	6.71E-01	-0.23	0.24	6.24E-01	-0.16	0.19	6.46E-01
phosphopantothenate biosynthesis III	-0.52	0.63	6.79E-01	-0.55	0.65	6.40E-01	-1.74	0.82	1.60E-01	-0.36	0.63	8.18E-01
CDP-archaeol biosynthesis	-0.50	0.61	6.79E-01	-0.54	0.63	6.38E-01	-1.65	0.80	1.68E-01	-0.35	0.62	8.15E-01
homolactic fermentation	0.04	0.04	6.81E-01	0.03	0.05	6.95E-01	-0.02	0.06	9.17E-01	0.04	0.04	6.62E-01
pyruvate fermentation to acetone	-0.10	0.13	6.84E-01	-0.08	0.13	7.55E-01	-0.58	0.16	5.62E-03	-0.09	0.13	7.68E-01
superpathway of sulfur oxidation (Acidianus ambivalens)	0.12	0.15	6.85E-01	0.20	0.15	4.29E-01	0.61	0.19	1.68E-02	0.12	0.15	7.08E-01
coenzyme B biosynthesis	-0.50	0.62	6.93E-01	-0.53	0.64	6.54E-01	-1.69	0.81	1.64E-01	-0.33	0.63	8.36E-01
L-tyrosine degradation I	-0.72	0.91	6.99E-01	-0.61	0.95	7.51E-01	-0.73	1.20	7.89E-01	-0.72	0.91	7.06E-01
coenzyme M biosynthesis I	-0.45	0.58	7.00E-01	-0.49	0.60	6.56E-01	-1.45	0.75	2.11E-01	-0.30	0.58	8.36E-01
sulfoglycolysis	0.62	0.80	7.02E-01	-0.48	0.83	7.89E-01	0.76	1.05	7.44E-01	0.62	0.80	7.08E-01
tetrahydromethanopterin biosynthesis	-0.48	0.62	7.07E-01	-0.52	0.64	6.58E-01	-1.57	0.81	2.07E-01	-0.32	0.63	8.40E-01
superpathway of L-phenylalanine biosynthesis	-0.08	0.10	7.07E-01	-0.14	0.11	4.14E-01	-0.06	0.13	8.39E-01	-0.08	0.10	7.08E-01
cob(II)yrinate a,c-diamide biosynthesis I (early cobalt insertion)	-0.17	0.22	7.08E-01	-0.36	0.23	3.04E-01	-0.19	0.29	7.63E-01	-0.16	0.22	7.36E-01

superpathway of taurine degradation	-0.55	0.72	7.10E-01	-0.52	0.74	7.23E-01	-1.14	0.93	4.88E-01	-0.44	0.72	7.96E-01
flavin biosynthesis II (archaea)	-0.49	0.64	7.10E-01	-0.53	0.66	6.69E-01	-1.64	0.83	2.01E-01	-0.33	0.65	8.39E-01
mevalonate pathway II (archaea)	-0.46	0.61	7.17E-01	-0.51	0.63	6.58E-01	-1.49	0.79	2.28E-01	-0.31	0.61	8.40E-01
superpathway of glycolysis and Entner-Doudoroff	-0.08	0.10	7.19E-01	-0.16	0.11	3.51E-01	-0.10	0.14	7.37E-01	-0.08	0.11	7.23E-01
superpathway of UDP-N-acetylglucosamine-derived O-antigen building blocks biosynthesis	-0.13	0.17	7.19E-01	-0.09	0.18	8.13E-01	-0.37	0.23	3.07E-01	-0.10	0.18	8.21E-01
tetrapyrrole biosynthesis II (from glycine)	0.05	0.06	7.22E-01	0.04	0.07	8.00E-01	0.13	0.08	3.57E-01	0.05	0.06	7.21E-01
UDP-N-acetyl-D-glucosamine biosynthesis I	0.05	0.07	7.34E-01	0.03	0.07	8.35E-01	0.03	0.09	9.02E-01	0.05	0.07	7.38E-01
superpathway of L-tyrosine biosynthesis	-0.07	0.10	7.40E-01	-0.13	0.11	4.46E-01	-0.06	0.13	8.42E-01	-0.07	0.10	7.47E-01
nitrate reduction VI (assimilatory)	0.09	0.13	7.48E-01	0.06	0.13	8.32E-01	0.32	0.17	2.05E-01	0.08	0.13	7.96E-01
superpathway of L-threonine metabolism	-0.44	0.64	7.51E-01	-1.14	0.66	2.63E-01	-0.16	0.83	9.40E-01	-0.46	0.64	7.37E-01
glycolysis I (from glucose 6-phosphate)	0.03	0.04	7.63E-01	0.03	0.04	7.55E-01	-0.02	0.05	8.96E-01	0.03	0.04	7.60E-01
3-phenylpropanoate degradation	-0.36	0.56	7.68E-01	-0.43	0.58	6.95E-01	-0.41	0.73	8.08E-01	-0.34	0.56	7.97E-01
D-galacturonate degradation I	0.04	0.07	7.72E-01	0.11	0.07	3.24E-01	0.00	0.09	9.99E-01	0.04	0.07	8.15E-01
superpathway of phenylethylamine degradation	-0.40	0.64	7.84E-01	-0.58	0.66	6.28E-01	-0.38	0.84	8.42E-01	-0.60	0.64	6.23E-01
myo-, chiro- and scillo-inositol degradation	0.08	0.13	7.84E-01	0.15	0.14	5.17E-01	0.13	0.17	7.44E-01	0.09	0.13	7.62E-01
phenylacetate degradation I (aerobic)	-0.40	0.65	7.84E-01	-0.60	0.67	6.22E-01	-0.39	0.85	8.42E-01	-0.66	0.65	5.92E-01
aspartate superpathway	0.04	0.07	7.84E-01	-0.03	0.07	8.48E-01	0.14	0.09	3.30E-01	0.04	0.07	7.91E-01
D-glucarate degradation I	0.11	0.18	7.95E-01	0.10	0.18	7.96E-01	0.12	0.23	8.23E-01	0.10	0.18	8.31E-01
tetrapyrrole biosynthesis I (from glutamate)	0.04	0.06	7.95E-01	0.03	0.06	8.58E-01	0.11	0.08	3.93E-01	0.04	0.06	7.96E-01
superpathway of N-acetylneuraminate degradation	0.05	0.08	7.98E-01	-0.02	0.08	9.26E-01	0.00	0.11	9.86E-01	0.05	0.08	7.82E-01
superpathway of hexitol degradation (bacteria)	0.06	0.10	8.01E-01	-0.02	0.10	9.38E-01	0.16	0.13	4.66E-01	0.06	0.10	8.15E-01

L-histidine degradation II	-0.33	0.58	8.08E-01	-0.35	0.60	7.79E-01	0.31	0.76	8.63E-01	-0.17	0.58	9.14E-01
L-lysine fermentation to acetate and butanoate	0.09	0.18	8.28E-01	0.14	0.18	6.73E-01	-0.50	0.23	1.51E-01	0.09	0.18	8.39E-01
2-methylcitrate cycle II	-0.32	0.68	8.55E-01	-1.21	0.70	2.69E-01	0.35	0.90	8.69E-01	-0.23	0.68	8.95E-01
mono-trans, poly-cis decaprenyl phosphate biosynthesis	0.19	0.40	8.55E-01	-0.08	0.41	9.38E-01	0.24	0.52	8.39E-01	0.19	0.40	8.53E-01
nitrate reduction I (denitrification)	-0.44	0.98	8.56E-01	-0.76	1.00	6.90E-01	-0.78	1.32	7.96E-01	-0.19	0.03	8.02E-07
formaldehyde assimilation II (RuMP Cycle)	0.09	0.19	8.56E-01	0.10	0.20	8.13E-01	0.32	0.25	4.44E-01	0.09	0.19	8.51E-01
thiazole biosynthesis I (E. coli)	0.02	0.05	8.56E-01	0.08	0.05	2.79E-01	0.02	0.06	9.05E-01	0.01	0.05	9.09E-01
6-hydroxymethyl-dihydropterin diphosphate biosynthesis I	-0.02	0.04	8.58E-01	-0.02	0.05	8.38E-01	0.02	0.06	8.88E-01	-0.02	0.04	8.33E-01
taxadiene biosynthesis (engineered)	-0.06	0.14	8.74E-01	-0.17	0.15	4.79E-01	-0.01	0.19	9.79E-01	-0.06	0.14	8.68E-01
pentose phosphate pathway	0.03	0.09	8.78E-01	0.03	0.09	8.63E-01	0.01	0.11	9.83E-01	0.03	0.09	8.81E-01
superpathway of L-tryptophan biosynthesis	-0.16	0.39	8.79E-01	-0.22	0.40	8.05E-01	0.09	0.51	9.43E-01	-0.15	0.39	8.80E-01
glycerol degradation to butanol	-0.09	0.23	8.80E-01	-0.13	0.24	8.00E-01	-0.23	0.31	7.37E-01	-0.06	0.24	9.20E-01
L-glutamate degradation V (via hydroxyglutarate)	-0.06	0.16	8.82E-01	-0.06	0.16	8.64E-01	-0.25	0.20	4.85E-01	-0.06	0.16	8.91E-01
4-deoxy-L-threo-hex-4-enopyranuronate degradation	-0.04	0.10	8.82E-01	0.04	0.10	8.62E-01	-0.17	0.13	4.57E-01	-0.05	0.10	8.54E-01
octane oxidation	0.09	0.27	8.91E-01	0.08	0.28	8.99E-01	0.25	0.35	7.45E-01	0.10	0.27	8.80E-01
ppGpp biosynthesis	-0.13	0.37	8.97E-01	-0.47	0.38	4.54E-01	-0.29	0.48	7.96E-01	-0.13	0.37	8.92E-01
mycothiol biosynthesis	0.19	0.58	9.02E-01	-0.15	0.60	9.23E-01	0.51	0.75	7.58E-01	0.19	0.58	9.00E-01
inosine-5'-phosphate biosynthesis III	0.05	0.16	9.13E-01	-0.13	0.16	6.62E-01	0.39	0.20	2.11E-01	0.04	0.16	9.25E-01
mevalonate pathway I	0.09	0.29	9.13E-01	-0.33	0.30	5.14E-01	0.77	0.38	1.79E-01	-0.02	0.29	9.83E-01
superpathway of geranylgeranyldiphosphate biosynthesis I (via mevalonate)	0.08	0.28	9.24E-01	-0.33	0.30	5.09E-01	0.74	0.37	1.89E-01	-0.02	0.29	9.80E-01
superpathway of tetrahydrofolate biosynthesis and salvage	0.01	0.03	9.24E-01	0.01	0.03	9.23E-01	0.02	0.04	8.06E-01	0.01	0.03	9.41E-01

superpathway of (R,R)-butanediol biosynthesis	0.05	0.22	9.28E-01	-0.18	0.22	6.55E-01	0.36	0.28	4.66E-01	0.00	0.22	9.96E-01
D-galactarate degradation I	0.06	0.28	9.34E-01	0.07	0.29	9.23E-01	0.12	0.36	8.96E-01	0.01	0.28	9.90E-01
superpathway of D-glucarate and D-galactarate degradation	0.06	0.28	9.34E-01	0.07	0.29	9.23E-01	0.12	0.36	8.96E-01	0.01	0.28	9.90E-01
acetyl-CoA fermentation to butanoate II	0.02	0.08	9.35E-01	0.05	0.09	8.01E-01	-0.14	0.11	4.71E-01	0.02	0.08	9.27E-01
hexitol fermentation to lactate, formate, ethanol and acetate	-0.02	0.14	9.47E-01	-0.06	0.15	8.54E-01	0.28	0.18	3.57E-01	-0.03	0.14	9.41E-01
L-tryptophan biosynthesis	0.01	0.03	9.50E-01	0.03	0.04	6.01E-01	-0.04	0.04	7.08E-01	0.00	0.03	9.73E-01
peptidoglycan biosynthesis V (β-lactam resistance)	0.04	0.26	9.62E-01	-0.25	0.27	5.90E-01	0.59	0.34	2.74E-01	-0.02	0.26	9.80E-01
gondoate biosynthesis (anaerobic)	0.00	0.03	9.64E-01	0.02	0.03	8.44E-01	0.02	0.04	8.69E-01	0.00	0.03	9.99E-01
GDP-mannose biosynthesis	-0.01	0.05	9.71E-01	0.00	0.05	9.93E-01	-0.15	0.06	8.15E-02	-0.01	0.05	9.61E-01
formaldehyde oxidation I	0.02	0.16	9.71E-01	0.03	0.17	9.38E-01	0.16	0.21	7.37E-01	0.02	0.17	9.71E-01
superpathway of glucose and xylose degradation	-0.01	0.09	9.80E-01	0.04	0.09	8.54E-01	-0.13	0.11	5.19E-01	-0.01	0.09	9.72E-01
pyrimidine deoxyribonucleotides de novo biosynthesis II	0.00	0.04	9.80E-01	-0.04	0.04	6.38E-01	-0.07	0.05	4.74E-01	0.00	0.04	9.84E-01
glycolysis II (from fructose 6-phosphate)	0.00	0.06	9.81E-01	-0.01	0.06	9.76E-01	-0.06	0.07	7.08E-01	0.01	0.06	9.64E-01
L-rhamnose degradation I	-0.01	0.09	9.82E-01	0.07	0.10	6.95E-01	-0.12	0.12	6.41E-01	-0.02	0.09	9.49E-01
superpathway of sulfolactate degradation	0.02	0.48	9.92E-01	0.03	0.50	9.90E-01	-0.81	0.63	4.55E-01	0.02	0.48	9.89E-01
2-methylcitrate cycle I	-0.03	0.78	9.92E-01	-1.29	0.81	3.04E-01	0.86	1.02	6.90E-01	-0.03	0.78	9.89E-01
NAD salvage pathway II	-0.01	0.20	9.92E-01	-0.28	0.21	4.04E-01	0.12	0.26	8.42E-01	0.00	0.21	9.99E-01
photorespiration	0.01	0.64	9.92E-01	0.06	0.66	9.73E-01	-1.08	0.83	4.49E-01	0.05	0.64	9.82E-01
superpathway of tetrahydrofolate biosynthesis	0.00	0.03	9.92E-01	-0.01	0.04	9.45E-01	0.02	0.04	8.92E-01	0.00	0.03	9.82E-01
CMP-legionaminate biosynthesis I	0.00	0.17	9.92E-01	0.00	0.18	9.95E-01	0.28	0.22	4.83E-01	-0.01	0.17	9.87E-01
cis-vaccenate biosynthesis	0.00	0.03	9.98E-01	0.01	0.03	8.81E-01	0.02	0.04	8.20E-01	0.00	0.03	9.72E-01

thiazole biosynthesis II (Bacillus)	0.00	0.12	9.98E-01	0.01	0.12	9.91E-01	0.10	0.15	7.66E-01	0.00	0.12	9.99E-01

[a] 10-year exposure window adjusted for sex, race, age, Parkinson's disease status, pesticides co-exposure, and sequencing platform.

[b] 6-to-10 years exposure window, aadjusted for sex, race, age, Parkinson's disease status, pesticides co-exposure, and sequencing platform.

[c] 0-to-5 years exposure window, aadjusted for sex, race, age, Parkinson's disease status, pesticides co-exposure, and sequencing platform.

[d] 10-year exposure window, adjusted for sex, race, age, pesticides co-exposure, and sequencing platform.

Abbreviations: Log2FC: Log2 Fold Change; SE: Standard Error: Adj: Adjusted.

Figure S1. Relative abundance plot at phylum level (Sorted by Firmicutes)

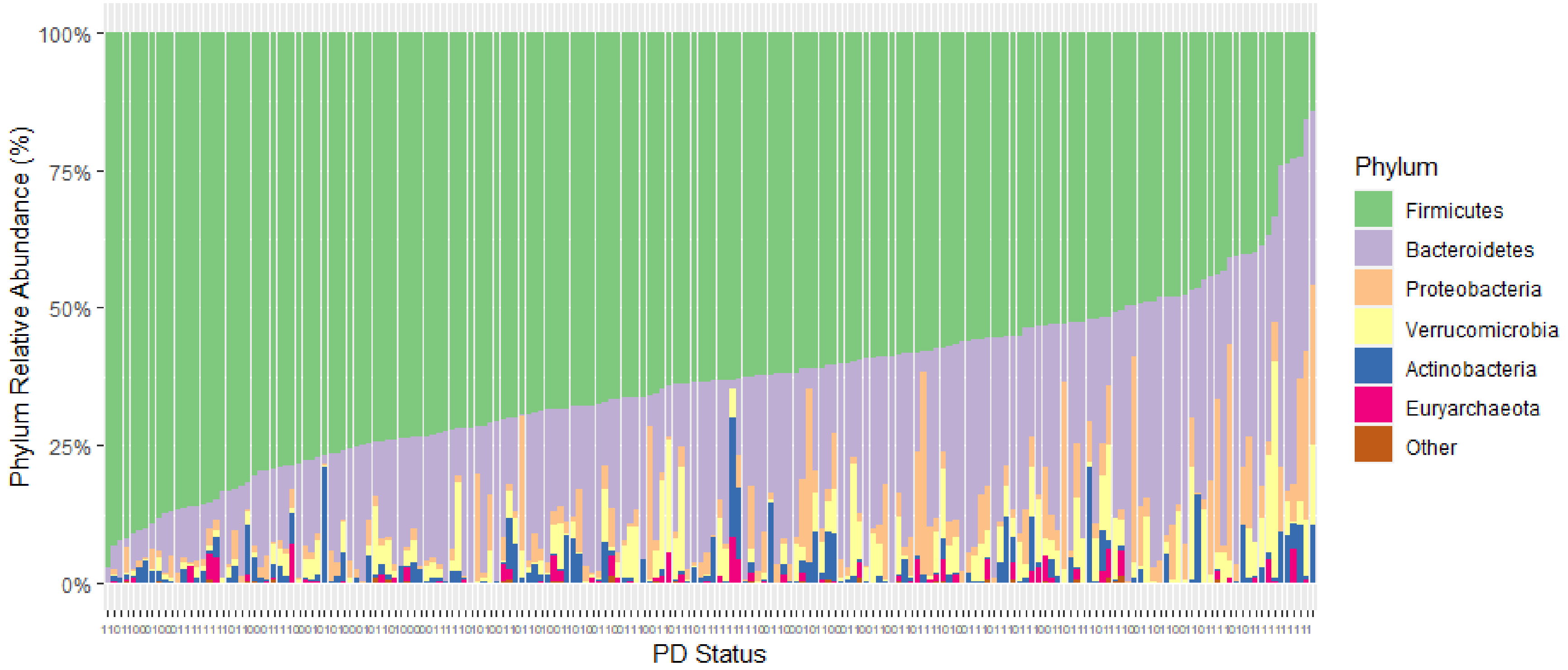

Figure S2. Averaged relative taxa abundance grouped by organophosphorus pesticides exposure

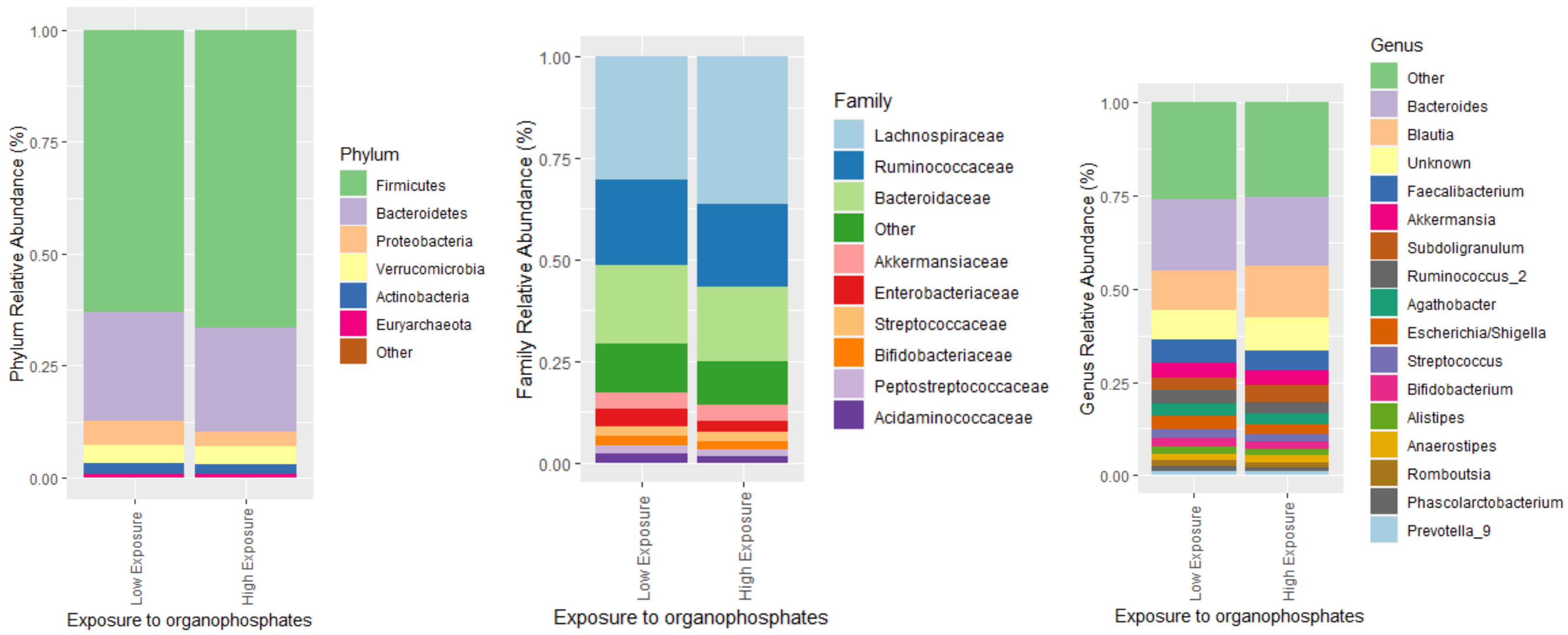

Left: Phylum level; Middle: Family level; Right: Genus Level

Figure S3. Exposure windows of main model and sensitivity models

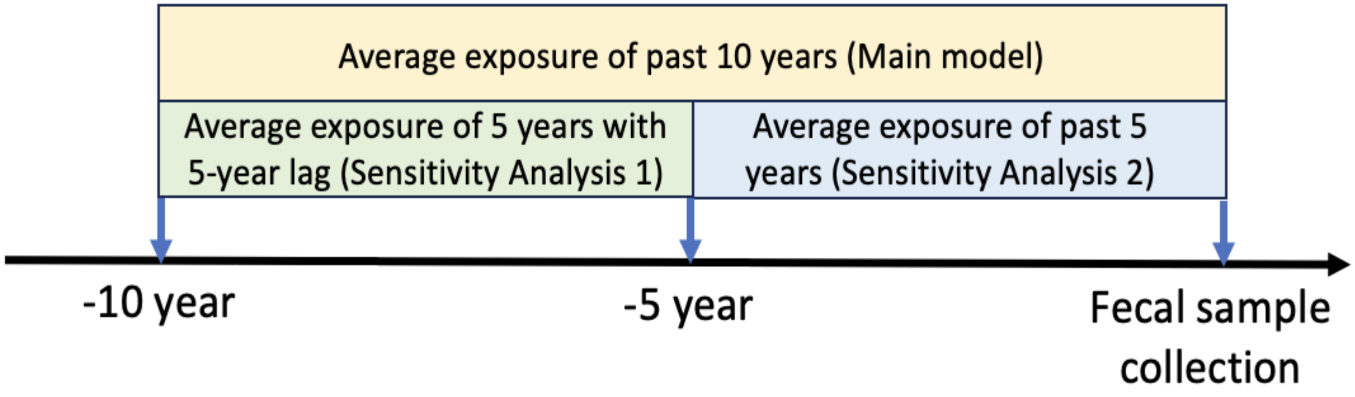

Figure S4. Distribution of organophosphorus pesticides exposure

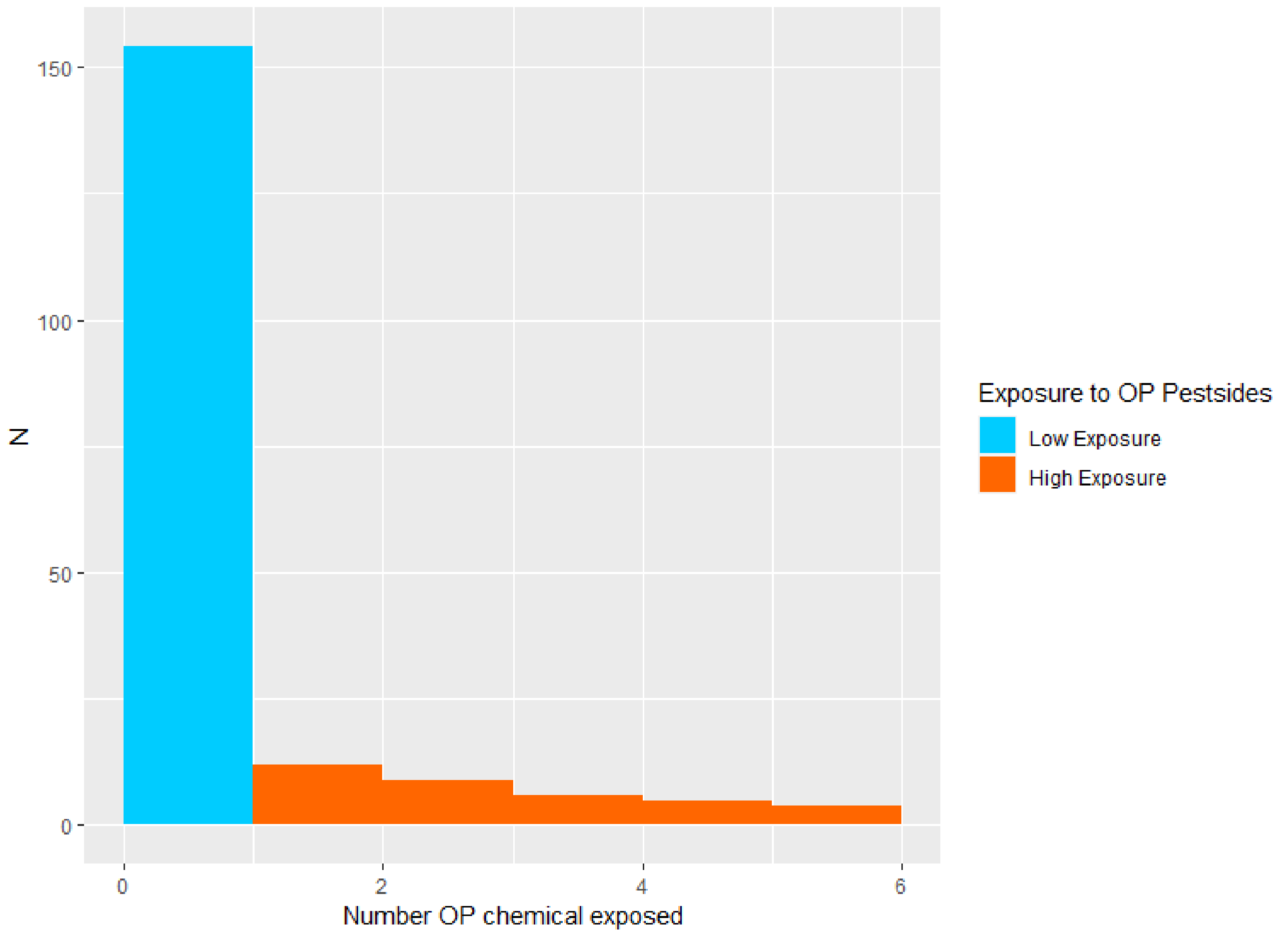

Figure S5. Comparison of microbiome profile between organophosphorus exposure groups

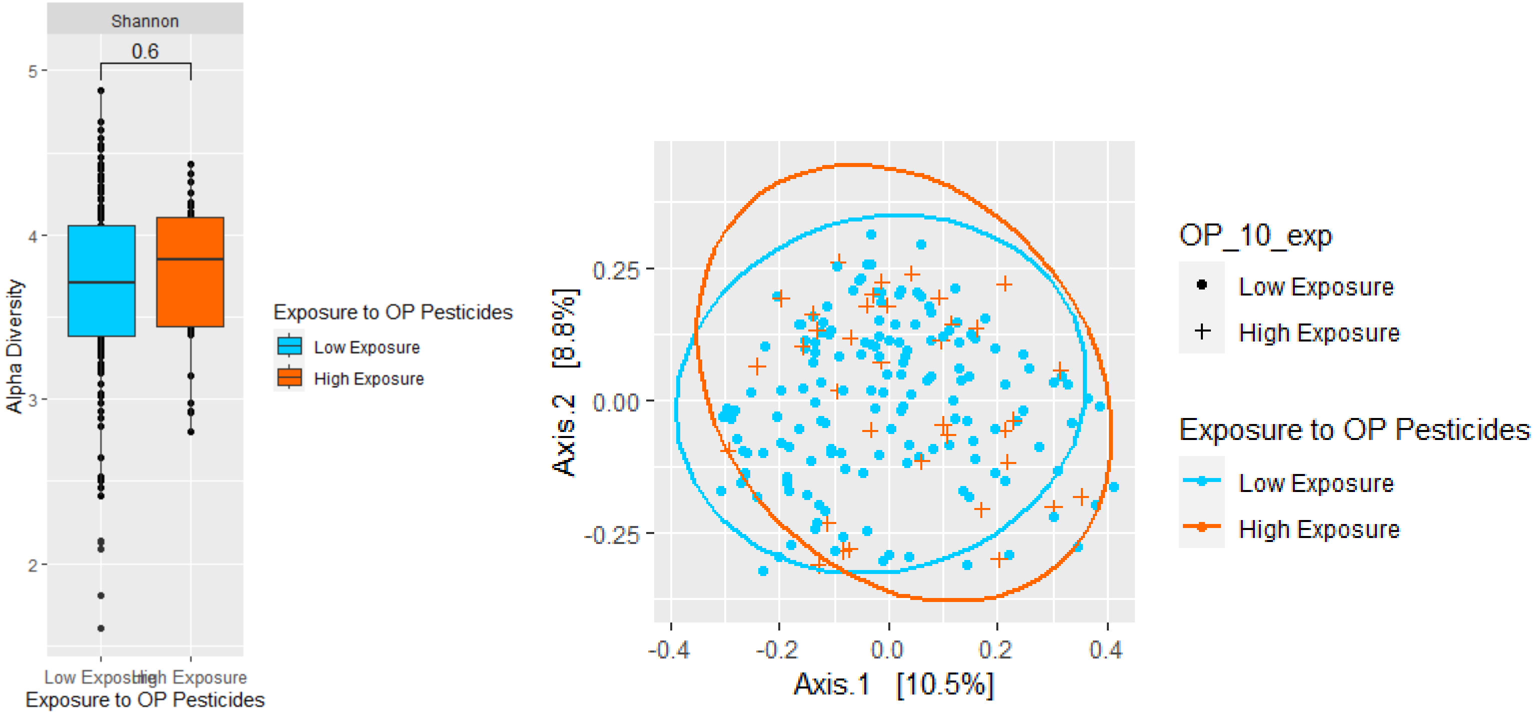

Figure S6. Bacterial taxa associated with organophosphorus pesticides exposure

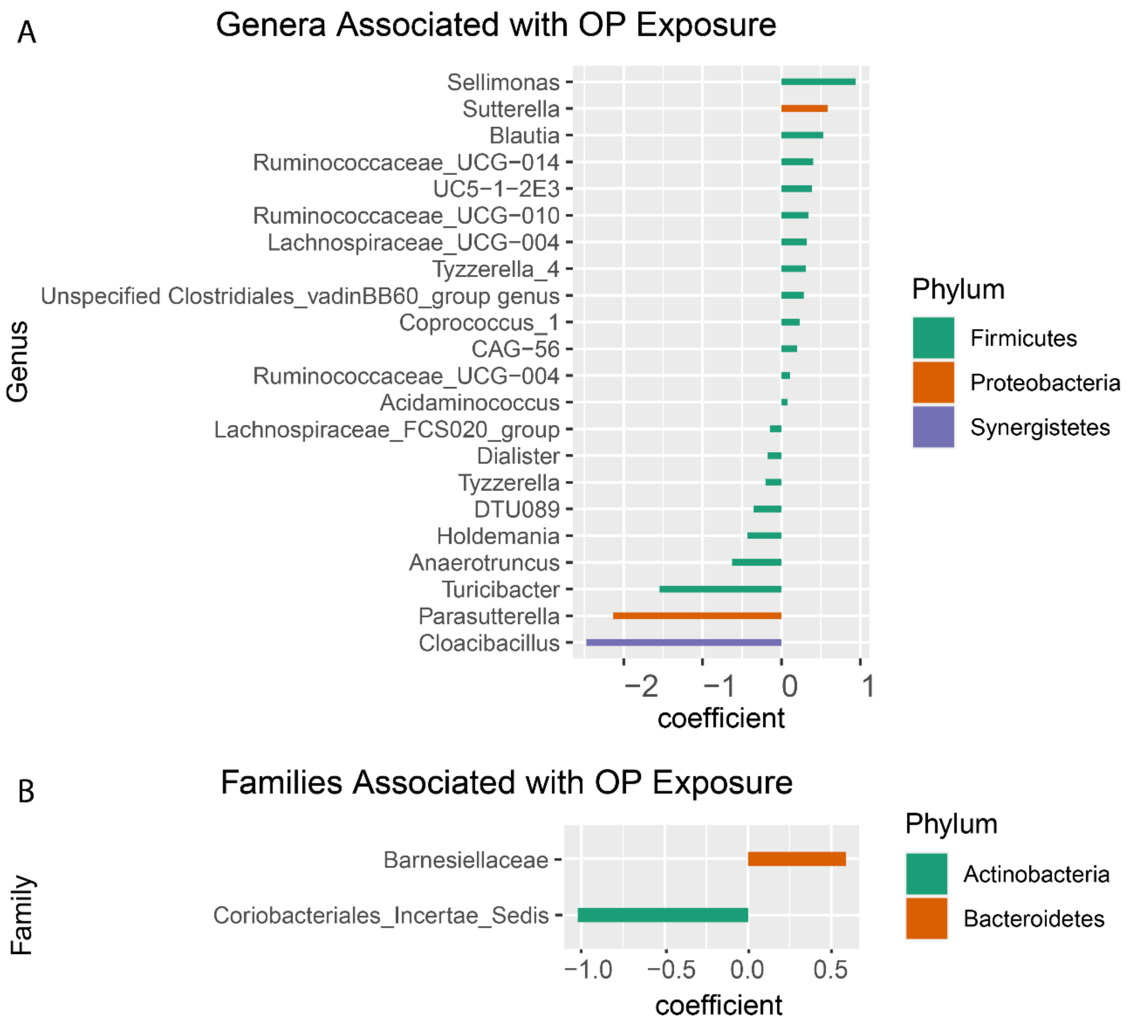

A. Differential taxa abundance at genus level
B. Differential taxa abundance at family level

Figure S7. Comparison of predicted metagene diversity between organophosphorus pesticides exposure groups

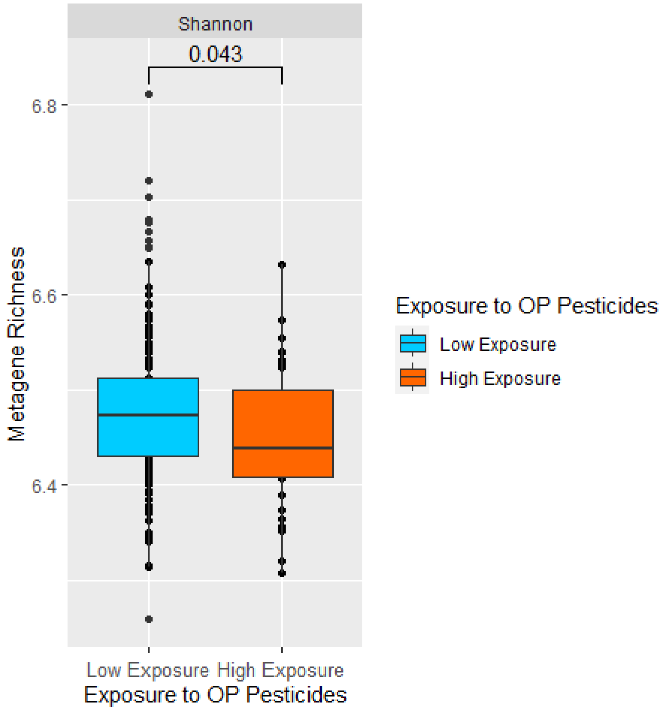

Figure S8. Predicted Metacyc pathways associated with organophosphorus pesticides exposure, grouped by level 2 superclasses

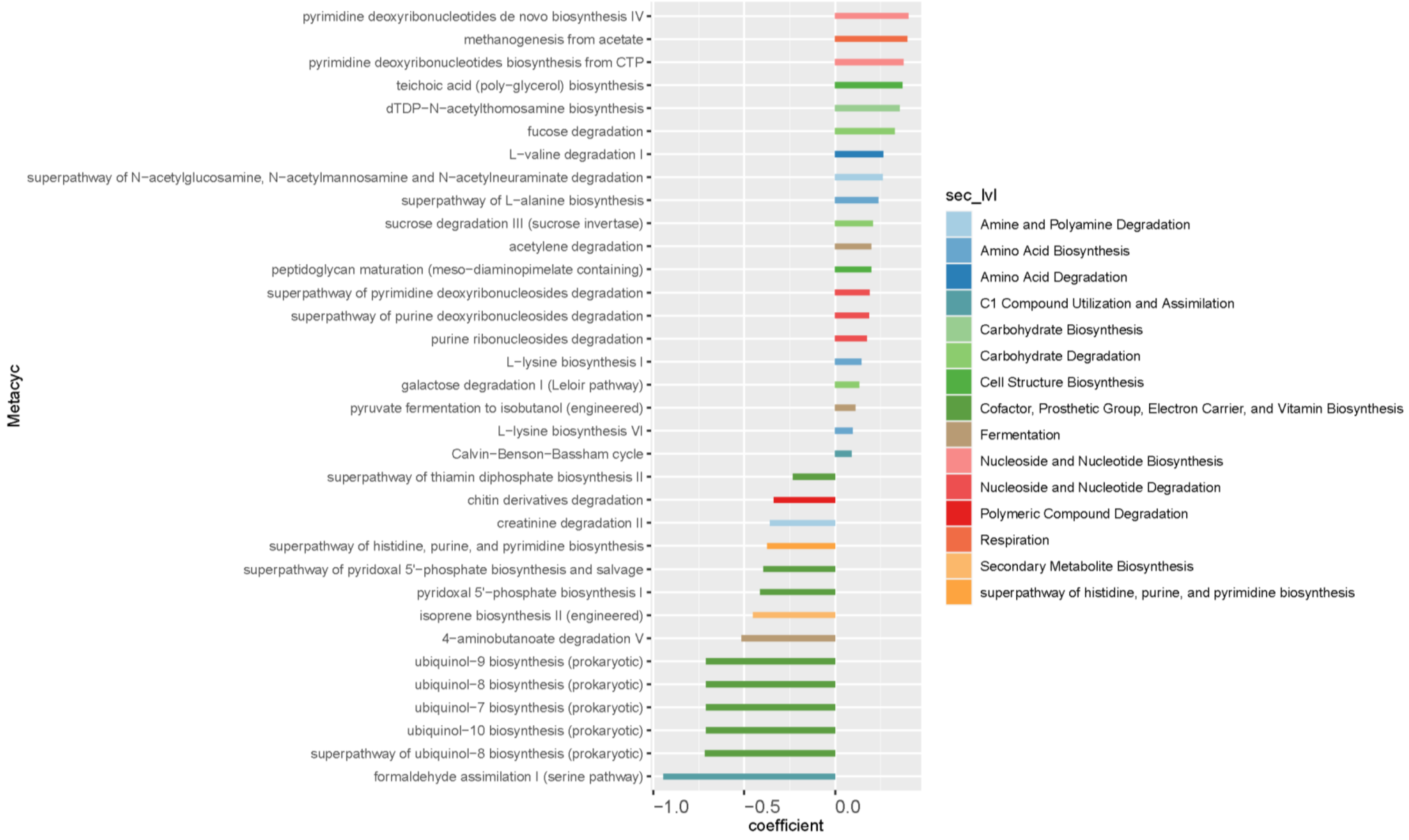

Figure S9. Predicted Metacyc associated with organophosphorus pesticides exposure, grouped by level 1 superclasses

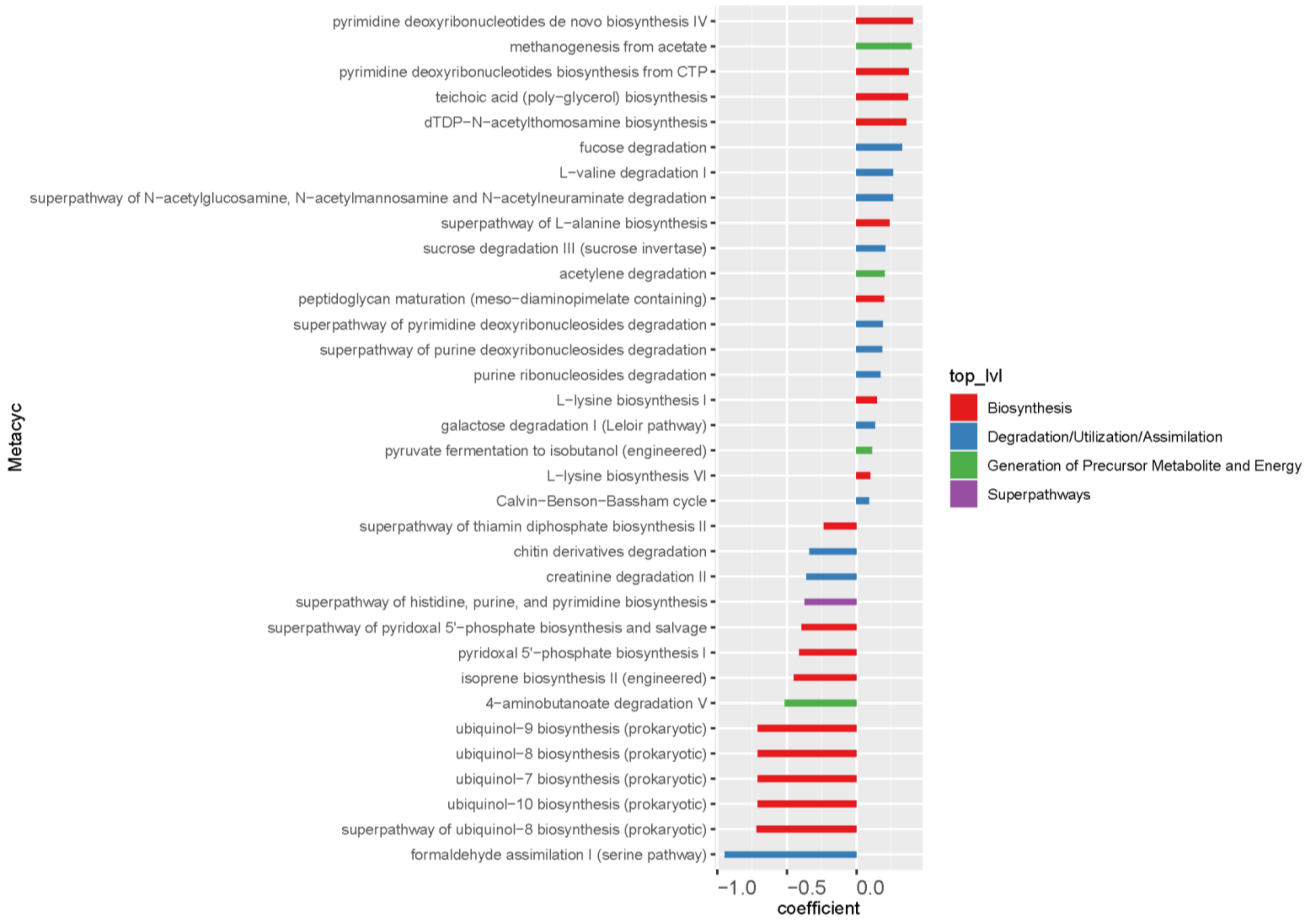

Supplement: Supplementary file 1 — Additional file 1: Table S1. List of other pesticide groups. Table S2. Differential taxa abundance associated with organophosphorus pesticides - Main model and sensitivity analyses - Phylum (N=190). Table S3. Differential taxa abundance associated with organophosphorus pesticides - Main model and sensitivity analyses - Family (N=190). Table S4. Differential taxa abundance associated with organophosphorus pesticides - Main model and sensitivity analyses - Genus (N=190). Table S5. Differential taxa abundance associated with organophosphorus pesticides - Main model and sensitivity analyses - predicted Metacyc pathways (N=190). Supplementary Figures: Figure S1. Relative abundance plot at phylum level (Sorted by Firmicutes). Figure S2. Averaged relative taxa abundance grouped by organophosphorus pesticide exposures. Figure S3. Exposure windows of main model and sensitivity analyses. Figure S4. Distribution of organophosphorus pesticide exposures. Figure S5. Comparison of microbiome profile between organophosphorus exposure groups. Figure S6. Bacterial taxa associated with organophosphorus pesticide exposures. Figure S7. Comparison of predicted metagene diversity between organophosphorus pesticids exposure groups. Figure S8. Predicted Metacyc pathways associated with organophosphorus pesticide exposure, grouped by level 2 superclasses. Figure S9. Predicted Metacyc pathways associated with organophosphorus pesticides exposure, grouped by level 1 superclasses [file 12940_2024_1078_MOESM1_ESM.pdf]
